# Experimental and DFT studies on the regioselective methanolysis of 5-azido-9-oxabicyclo[6.1.0]nonan-4-yl 4-nitrobenzoate isomers

**DOI:** 10.3762/bjoc.22.40

**Published:** 2026-03-26

**Authors:** İlknur Polat, Selçuk Eşsiz, Emine Salamci

**Affiliations:** 1 Department of Chemistry, Faculty of Sciences, Atatürk University, 25240 Erzurum, Türkiye; 2 Hakkari University, Vocational School of Health Services, Department of Medical Services and Techniques, 30000 Hakkari, Türkiye

**Keywords:** azides, 8-azidocyclooct-4-en-1-yl 4-nitrobenzoate, DFT, epoxycyclooctane azide, methanolysis

## Abstract

The regioselective methanolysis of new azido-4-nitrobenzoate epoxycyclooctane isomers and the characterization of the resulting products are described herein. Firstly, treatment of key compound 8-azidocyclooct-4-en-1-ol with 4-nitrobenzoyl chloride followed by an epoxidation reaction and then methanolysis of the epoxide ring and acetylation resulted in the formation of two corresponding chloro-acetate isomers. The structure of one of the chloro-acetate isomers was determined via crystallographic analysis and the other by 1D and 2D NMR spectroscopy. DFT computations confirm the regioselectivity of the methanolysis process, highlighting its precision and efficiency.

## Introduction

**Figure 1:**
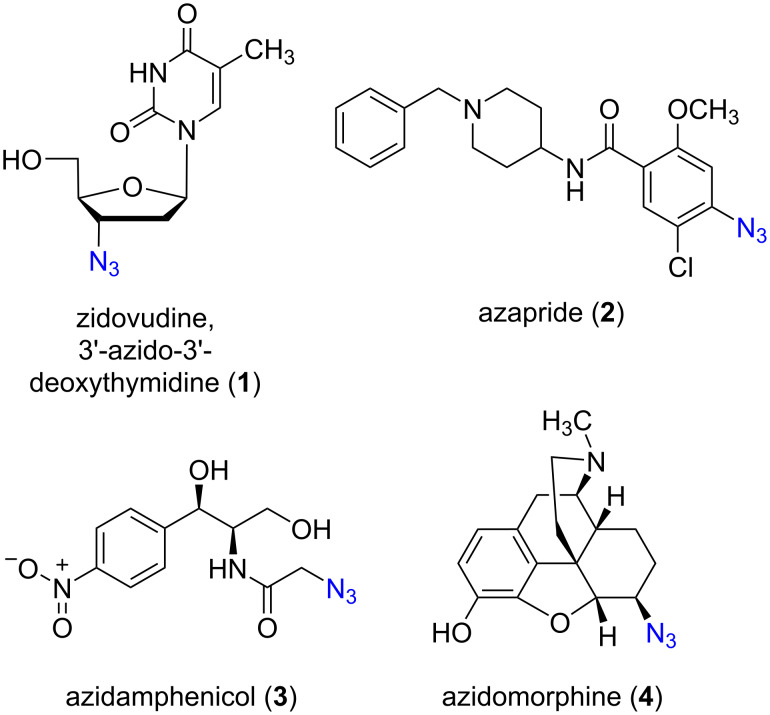
Some important bioactive molecules with an azide group.

Organic azides are very important precursors for the preparation and synthesis of various nitrogen-containing compounds, as well as triazoles and aminocyclitols, which have numerous applications in industry, medicine, and pharmacy [1–5]. They are found in many biologically active compounds. For example, 3’-azido-3’-deoxythymidine (zidovudine, **1**) is used in treatment of AIDS, while azapride (**2**), azidamphenicol (**3**), and azidomorphine (**4**) are utilized as pharmaceuticals (Figure 1) [1,6–9]. Additionally, recent studies demonstrated that nonglycosyl azides hamper glycan acceptance by carbohydrate-processing enzymes [10–11]. Moreover, azido sugars are used directly in cell biology studies. Therefore, synthetic methodologies for the preparation of this class of compounds are of considerable interest.

Azides have traditionally been used as protected primary amine equivalents. Therefore, in our previous studies, we employed azides containing eight-membered rings as precursors in the synthesis of C8-aminocyclitols [12–19].

The ring-opening reactions of bicyclic epoxides are of significant importance and scientific interest due to their high ring strain, their strategic role in the construction of complex molecular architectures, and their utility in the synthesis of natural products, pharmaceutical compounds, and chiral ligands. The Lewis acid-catalyzed ring-opening reaction of cyclohexene oxide by MeZH (Z = O, S, and NH) is well known [20]. However, studies on large-sized bicyclic epoxides remain relatively limited in the published literature. In our previous study, we performed the ring-opening reaction of an azidoepoxide containing an eight-membered ring with HCl(g) in MeOH by regioselectivity [12].

More recently we have directed our interest towards developing an efficient method for the synthesis of new C8-azides. In the present paper, we report the first synthesis of 5-azido-9-oxabicyclo[6.1.0]nonan-4-yl 4-nitrobenzoate isomers starting from *cis,cis*-1,5-cyclooctadiene.

## Results and Discussion

**Scheme 1:**
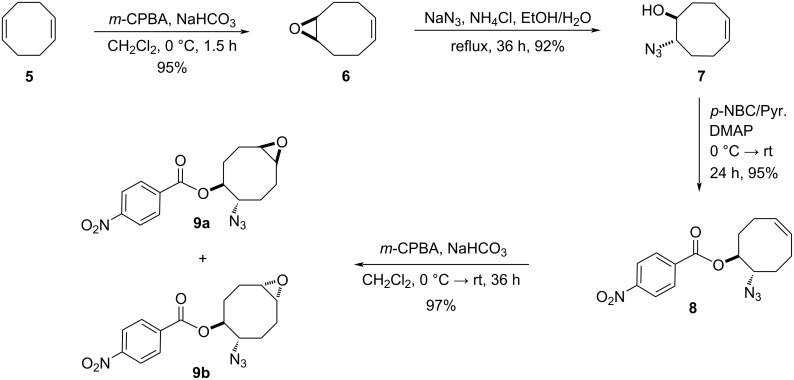
Epoxidation of azido nitrobenzoate **8**.

For the synthesis of the hitherto unknown key compound 8-azidocyclooct-4-en-1-yl 4-nitrobenzoate (**8**), we commenced by obtaining the known 5,6-epoxycyclooctene (**6**), which was prepared by epoxidation of *cis,cis*-1,5-cyclooctadiene (**5**) with *m*-CPBA [21–22]. Subsequently, the 5,6-epoxycyclooctene (**6**) was reacted with NaN_3_ and NH_4_Cl in EtOH/H_2_O to give azidol **7** in 92% yield [23] (Scheme 1). Azidol **7** was then converted into the corresponding azido nitrobenzoate **8** with *p*-nitrobenzoyl chloride (*p-*NBC) in pyridine and 4-(dimethylamino)pyridine (DMAP) in 95% yield. Treatment of azido nitrobenzoate **8** with *m*-CPBA gave a mixture of isomeric epoxides **9a** and **9b** with a combined yield of 97%. The formation and ratio of these isomers were determined by NMR spectroscopy, showing a 63:37 ratio (^1^H NMR) of **9a** and **9b**, respectively, but all attempts to separate the isomeric epoxides **9a** and **9b** by column chromatography or crystallization failed.

Eight-membered rings are known to preferentially adopt boat-chair conformations, which minimize torsional and transannular strain [24]. To further rationalize the observed regioselectivity, steric drawings of epoxides **9a** and **9b** were constructed using a bathtub-like representation of the cyclooctane ring (Table 1). For epoxide **9a**, the majority of dihedral angles fall within the ±60–80° range, consistent with a regular boat-chair geometry. The limited deviation from ideal gauche values and the absence of severely over-twisted dihedrals indicate a relatively low-strain conformation. In contrast, epoxide **9b** exhibits a more distorted dihedral distribution, including a strongly twisted dihedral (≈90°) together with unevenly distributed gauche and near-planar torsions. This pattern is consistent with a less favourable, distorted boat-chair (or twist-boat-chair-like) conformation, associated with increased torsional strain, thereby disfavouring formation of **9b** relative to **9a**.

**Table 1:** Dihedral angles (τ, °) in the optimized structure of epoxides **9**.

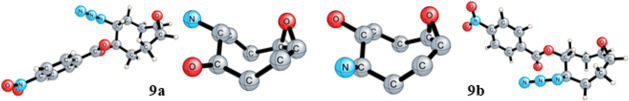								

Entry	τ_1_	τ_2_	τ_3_	τ_4_	τ_5_	τ_6_	τ_7_	τ_8_

**9a**	−81.0	45.8	64.2	−67.2	−1.4	−20.7	82.3	−20.5
**9b**	−67.5	−43.9	89.5	−14.4	−2.5	−64.7	53.0	55.1

**Scheme 2:**
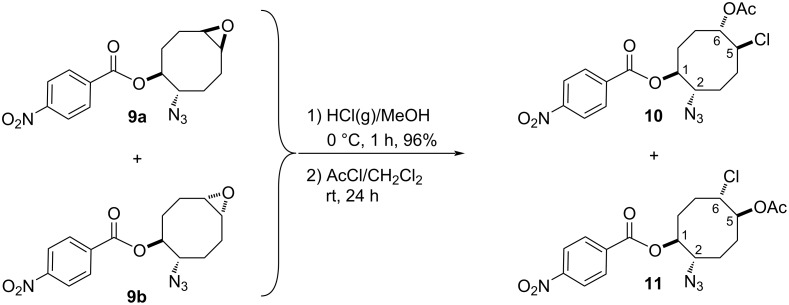
Methanolysis of the mixture of isomeric epoxides **9a** and **9b**.

**Figure 2:**
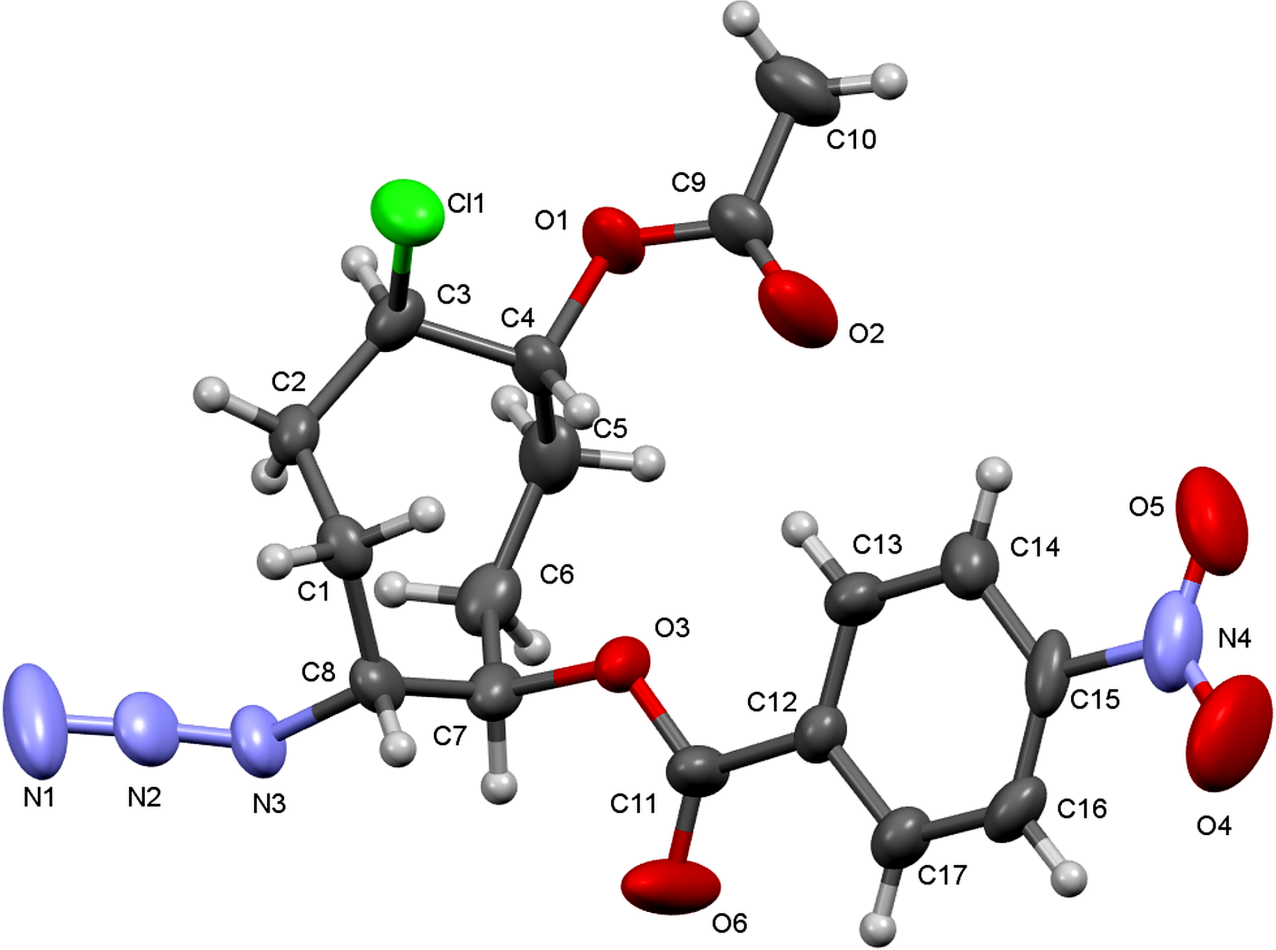
The X-ray crystal structure of **10**.

Next, we turned our attention to the methanolysis of the mixture of isomeric epoxides **9**. A ring-opening reaction of the epoxides **9** with HCl(g) in MeOH resulted in the formation of chlorohydrin-azidobenzoate isomers **10** and **11** in a total yield of 96% (Scheme 2). Then, for full characterization of the structures of the obtained products, the mixture of chlorohydrins was converted into the corresponding acetates **10** and **11** using AcCl in CH_2_Cl_2_. The reaction mixture was chromatographed on a silica gel column with *n*-hexane/ethyl acetate 85:15 as eluent to give pure acetates **10** and **11** in 57% and 35% yields, respectively. The structure of compound **10** was unambiguously determined by single crystal X-ray analysis (Figure 2) [25]. X-ray analysis of azido compound **10** confirmed the *trans* configuration of the azide group with chloride and the *cis* configuration with the acetate.

**Scheme 3:**
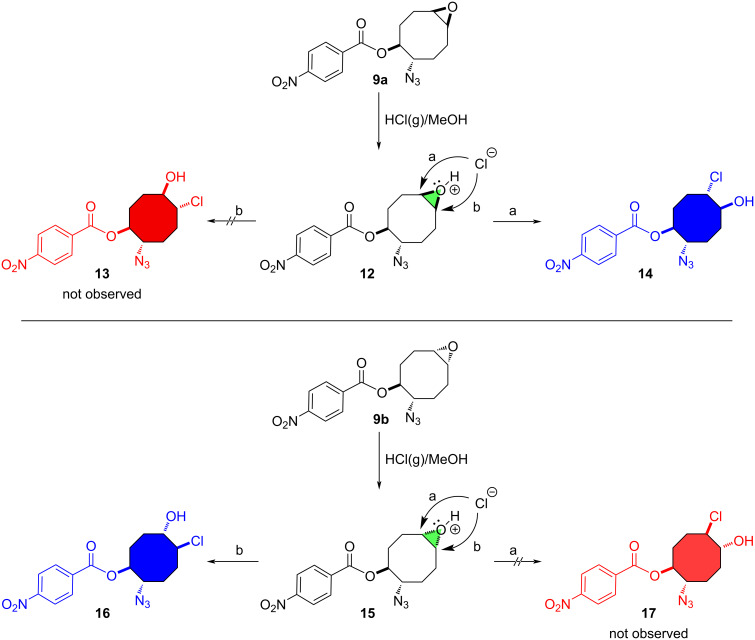
Suggested mechanism for the reaction of the isomeric epoxides **9a** and **9b** with HCl(g) in MeOH.

Our suggested mechanism for the ring-opening of the epoxides **9** with HCl(g) in MeOH proceeded as described in Scheme 3. As shown, the *syn*-face of the epoxide isomers **9a** and **9b** with the benzoate in **9a** and the azide in **9b** is more blocked than the *anti*-face. Therefore, the sterically hindering effect of these groups is observed in the *syn*-face. We assume that the chloride anion prefers to first attack the epoxide ring of intermediate **12** with the *anti*-face of the benzoate to give compound **14**. Similarly, the chloride anion attacks by S_N_2-type the epoxide ring of intermediate **15** to give the sole product **16**.

The structure of acetate **11** was investigated using 1D (^1^H and ^13^C) and 2D (COSY, NOESY, NOE-diff, and HMQC) NMR spectroscopic data. However, the uncertainty or weakness of the cross peaks in NOESY and NOE signals indicated that compound **11** exhibits significant conformational flexibility. The cyclooctane ring has increased flexibility, so the configuration assignment of **11** is difficult to determine with the help of the NOESY experiments or the other 2D NMR conducted. Compound **11** is not a single crystal, so the determination by X-ray analysis of its structure was not possible. We strongly assume that the structure of **11** is based on the methanolysis mechanism of the epoxide **9a** (Scheme 3).

In order to elucidate the formation mechanisms of the products yielded from the methanolysis of isomeric epoxides **9**, we performed a series of density functional theory (DFT) computations using the software Gaussian 16 [26]. For this, geometry optimizations and harmonic vibrational frequency computations for the structures considered were carried out with DFT at the M06-2X hybrid functional [27] augmented by Grimme’s dispersion correction DFT-D3 to improve the description of long-range dispersion interactions [28]. For this purpose, a Pople-type polarized triple-ζ split valence basis set with diffuse functions on all atoms, 6-311G++(d,p) [29–31], and SMD [32] model (methanol (ε = 32.613)) were employed. Initial geometries were pre-optimized using MM2 (ChemDraw 3D) prior to DFT optimization to account for conformational flexibility. The 3D molecular structures were visualized using the open-source software cheMVP.exe.

**Figure 3:**
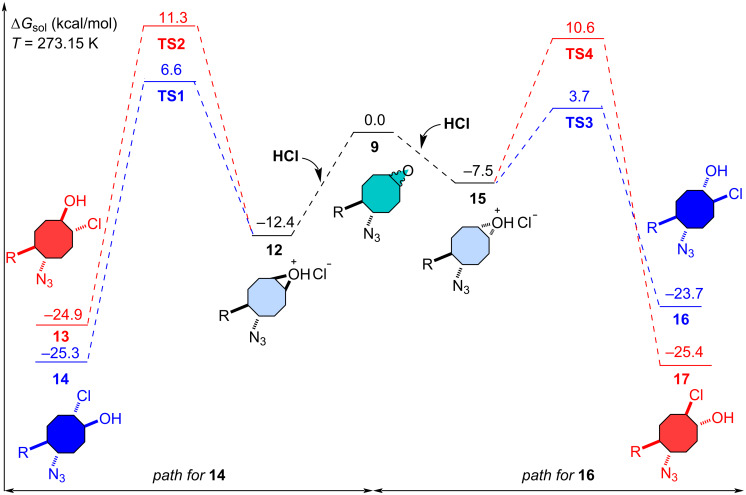
Relative free energy profile for the methanolysis of the isomeric epoxides **9**.

DFT computations were performed to gain insights into the mechanism underlying regioselectivity for the methanolysis of the isomeric epoxides **9** (Figure 3). Detailed mechanistic routes involving all possible intermediates responsible for the regioselectivity step were constructed and the free energy barriers for each elementary reaction step along these routes were computed. The acid-mediated ring-opening reaction in the isomeric epoxides **9**, initiated by protonation, can follow two possible pathways – either along the intermediate **12** or along the intermediate **15**. There is a possibility of two products being formed in both pathways. Free energy computations indicate that the intermediate **12** is thermodynamically more stable by 4.9 kcal mol^−1^ compared to intermediate **15**. The nucleophilic attack by chloride ion bifurcates into two paths, namely C1- (route a) and C2-attacks (route b) for intermediates **12** and **15**. Halohydrin **14** is yielded with a C1-attack on intermediate **12**, while **13** is yielded with a C2-attack. For the formation of halohydrin **14**, **12** → **14**, the computed reaction and activation free energies are −12.9 and 19.0 kcal mol^−1^, respectively, while for the **12** → **13** conversion, the computed reaction and activation free energies are −12.5 and 23.7 kcal mol^−1^, respectively. Halohydrin **16** is yielded by a C2-attack on **15**, whereas a C1-attack yields **17**. For the formation of halohydrin **16**, **15** → **16**, the computed reaction and activation free energies are −16.2 and 11.2 kcal mol^−1^, respectively, while for **15** → **17** conversion, the computed reaction and activation free energies are −17.9 and 18.1 kcal mol^−1^, respectively. The DFT results are rationally explained and supported our experimental observations, indicating that the methanolysis of both epoxides **9a** and **9b** preferentially follows the kinetically favourable pathway.

The conformational preferences of the cyclooctane ring were analyzed based on the eight consecutive dihedral angles (τ_1_–τ_8_, °), which are summarized in Table 2, following the classification proposed by Pakes and co-workers [24]. TS1 displays a predominantly boat-chair geometry, with only one over-twisted dihedral angle (τ_3_ = 94°), indicating a moderately distorted boat-chair conformation. In contrast, TS2 exhibits a characteristic twist-boat-chair arrangement, evidenced by the co-existence of a strongly over-twisted segment (τ_2_ = 101.8°) and a nearly planar dihedral (τ_6_ = 4.0°). A similar trend is observed for TS3 and TS4. TS3 retains a largely boat-chair-like geometry, however, the presence of a single over-twisted dihedral (τ_7_ = 93.3°) indicates the onset of conformational distortion. By contrast, TS4 exhibits multiple over-twisted dihedrals (τ_2_ = 106.1° and τ_7_ = 104.0°, τ_3_= −88.2°) together with a nearly planar dihedral (τ_5_ = −15.5°), unambiguously identifying this structure as a strongly distorted twist-boat-chair conformation. Overall, these results demonstrate that progression along the reaction coordinate is accompanied by increasing torsional strain within the cyclooctane ring, which correlates well with the computed activation barriers and the divergence of the competing reaction pathways. In this context, despite the presence of a hydrogen-bonding interaction in TS2, TS1 is lower in energy due to a more favourable cyclooctane conformation. Notably, the relatively small energy difference between TS1 and TS2 (4.7 kcal/mol), compared to that between TS3 and TS4 (6.9 kcal/mol), can be attributed to hydrogen-bond stabilization in TS2, indicating that conformational effects play a more dominant role than hydrogen bonding in determining transition-state stability.

**Table 2:** Dihedral angles (τ, °) in the optimized transition states.

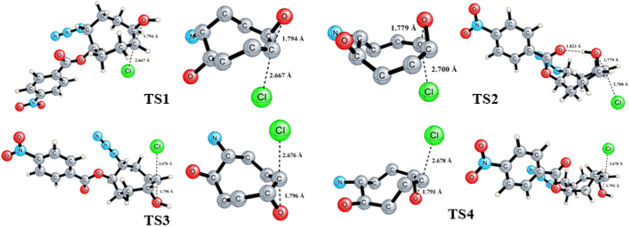									

TS	τ_1_	τ_2_	τ_3_	τ_4_	τ_5_	τ_6_	τ_7_	τ_8_	conformer

TS1	−67.8	−48.3	94.0	−30.9	22.6	−74.7	40.0	68.8	boat-chair
TS2	−37.9	101.8	−62.9	22.0	−28.7	4.0	70.9	−66.3	twist-boat-chair
TS3	−67.0	62.5	44.4	−76.6	22.5	−32.3	93.3	−45.3	boat-chair
TS4	−61.9	106.1	−88.2	62.8	−15.5	−67.2	104.0	−27.2	twist-boat-chair

**Figure 4:**
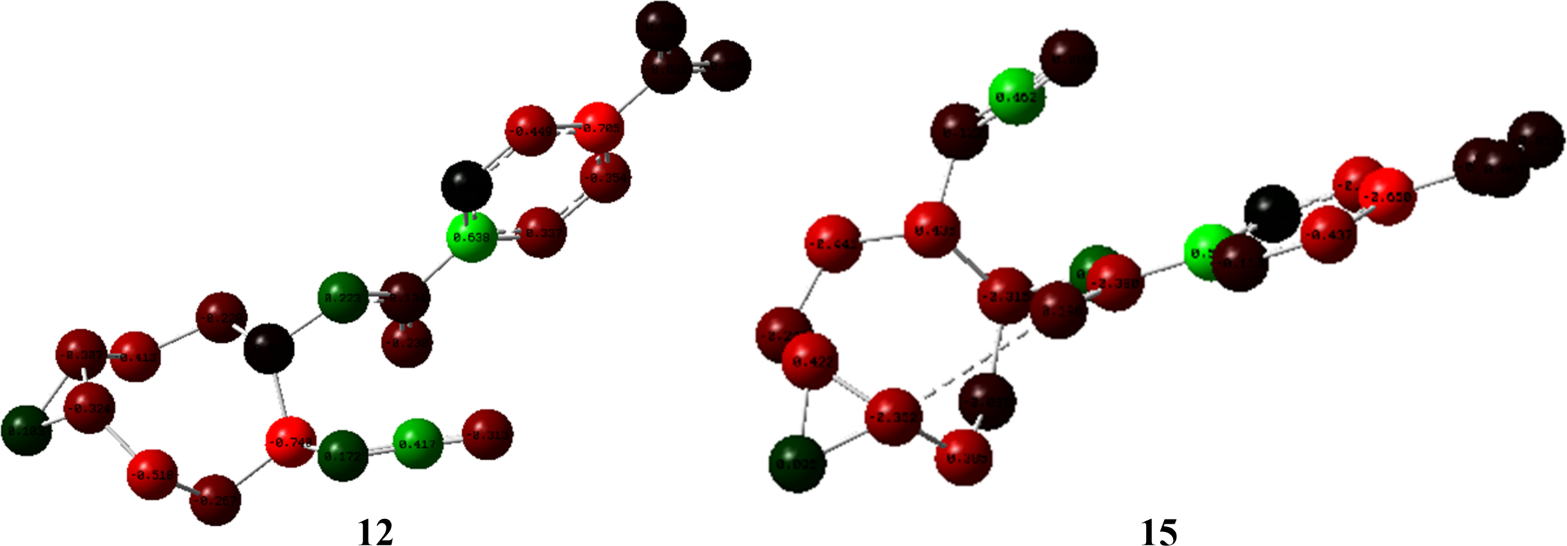
Mulliken charge analysis of protonated epoxide intermediates **12** and **15**.

Mulliken charge analysis for the protonated epoxide intermediates **12** and **15**, derived from epoxides **9a** and **9b**, respectively, reveals that the charge differences between the epoxide carbons are not pronounced for either diastereomer, indicating that electronic effects alone are insufficient to explain the observed regioselectivity (Figure 4). In the protonated intermediate **12** derived from epoxide **9a**, the carbon atom preferentially attacked by chloride bears a relatively more positive charge, which is consistent with an electronically favoured nucleophilic attack. In contrast, for the protonated intermediate **15** derived from epoxide **9b**, the more positively charged epoxide carbon is sterically shielded by the neighbouring ester carbonyl group. As a result, nucleophilic attack occurs at the sterically more accessible, albeit less positively charged, epoxide carbon. These observations indicate that the regioselectivity of epoxide **9a** is governed by a combination of electronic and steric factors, while steric effects are more pronounced for epoxide **9b**.

## Conclusion

We achieved the first synthesis of new cyclooctane-azide derivatives (**8**, **10**, and **11**) efficiently from commercially readily available *cis,cis*-1,5-cyclooctadiene. The key step, the ring opening of epoxide **9**, proceeded smoothly by methanolysis with HCl(g) in MeOH. The regioselectivity of oxirane-ring opening in **9** was attributed to the conformational effects. The mechanism for the formation of compounds **10** and **11** was elucidated with DFT computations. TS1 and TS3 predominantly retain boat-chair-like geometries, whereas TS2 and especially TS4 adopt significantly distorted twist-boat-chair conformations. The associated torsional strain accounts for the increased activation barriers and favours regioselectivity. These novel compounds synthesized may be used as precursors to design pharmacological tools in the future.

## Experimental

### General information

Melting points are uncorrected. Infrared spectra were obtained from solution in 0.1 mm cells or KBr pellets on an FT-IR Mattson 1000 instrument. The ^1^H and ^13^C NMR spectra were recorded on 400 (100) MHz Varian or 400 (100) MHz Bruker spectrometers and are reported in δ units with SiMe_4_ as internal standard. HRMS spectra were obtained on a Bruker microTOF-Q or Agilent 6530 Accurate Mass Q-TOF instrument. Melting points were determined on a GallenKamp MPD 350. Column chromatography was performed on silica gel (60 mesh, Merck). TLC was carried out on Merck 0.2 mm silica gel 60 F_254_ analytical aluminium plates.

### (1R*,8S*,*Z*)-9-Oxabicyclo[6.1.0]non-4-ene (**6**)


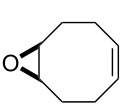


A magnetically stirred solution of *cis,cis*-1,5-cyclooctadiene (**5**, 1.00 g, 9.24 mmol) in 50 mL dichloromethane was cooled to 0 °C. *m*-CPBA (2.20 g, 77%, 9.82 mmol) and NaHCO_3_ (0.78 g, 9.29 mmol) were added to the solution and stirred for 1.5 hours. Subsequently, 50 mL of 1.5 M NaOH solution was added and the reaction mixture was stirred for 15 min at 0 °C, then extracted with dichloromethane (4 × 30 mL). The organic layer was dried over Na_2_SO_4_, filtered, and evaporated under reduced pressure. The crude product was purified by silica gel column chromatography eluting with pure hexane to give as the first fraction monoepoxide **6** (1.09 g, 8.78 mmol, 95%, a colourless oil) and as the second bisepoxide (0.07 g, 0.5 mmol, 5%, a white solid). The spectroscopic data are in accordance with those reported in the literature [22].

### (1S*,8S*,*Z*)-8-Azidocyclooct-4-en-1-ol (**7**)


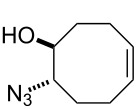


Cyclooctene monoepoxide **6** (2.00 g, 16.11 mmol) was dissolved in a 3:2 mixture of ethanol/water (80 mL). NaN_3_ (6.28 g, 96.60 mmol) and NH_4_Cl (1.72 g, 32.16 mmol) were added and the reaction mixture was refluxed. The progress was monitored by TLC and the reaction was completed in 36 hours. The solvent was removed by rotary evaporation, 30 mL water was added to the residue and the mixture was extracted with ethyl acetate (4 × 25 mL). The combined organic phases were dried over Na_2_SO_4_ and concentrated in vacuo. Purification by silica gel column chromatography eluting with EtOAc/hexane 5:95 provided azidol **7** [23] as slightly yellow oil (2.47 g, 14.77 mmol, 92%, lit. [23] 71%). ^1^H NMR (400 MHz, CDCl_3_) δ 5.69–5.61 (m, 1H), 5.60–5.52 (m, 1H), 3.79–3.65 (m, 2H), 2.56–2.36 (m, 2H), 2.27–2.09 (m, 4H), 1.81–1.67 (m, 2H); ^13^C NMR (100 MHz, CDCl_3_) δ 130.3, 127.5, 73.1, 66.8, 32.4, 29.7, 23.5, 23.1.

### (1S*,8S*,*Z*)-8-Azidocyclooct-4-en-1-yl 4-nitrobenzoate (**8**)


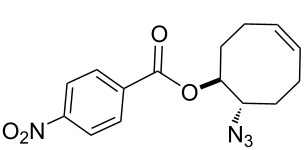


The azidol **7** (0.50 g, 2.99 mmol) was dissolved in 9 mL of anhydrous pyridine and the solution was cooled to 0 °C. *p*-Nitrobenzoyl chloride (1.11 g, 5.98 mmol) and 4-(dimethylamino)pyridine (DMAP, 2 mg) were added and the mixture was stirred at room temperature for 24 hours. Then, the solution was cooled to 0 °C and 100 mL of 2 M HCl added and stirred for 5 min. The mixture was extracted with ethyl acetate (4 × 30 mL). The organic phase was washed with saturated NaHCO_3_ (2 × 25 mL) and water (2 × 30 mL), dried over Na_2_SO_4_, and concentrated in vacuo. The crude product was purified by chromatography on a silica gel column eluting with EtOAc/hexane 20:80 to give pure azido benzoate **8** (0.90 g, 2.85 mmol, 95%). The azido benzoate **8** was recrystallized from EtOAc/hexane to give slightly yellow crystals; mp 60–62 °C. ^1^H NMR (400 MHz, CDCl_3_) δ 8.29–8.19 (m, 4H, aromatic), 5.72–5.59 (m, 2H, H-4 and H-5), 5.36–5.27 (m, 1H, H-1), 3.96 (td, *J* = 9.2, 3.7 Hz, 1H, H-8), 2.60–1.71 (series of m, 8H, CH_2_); ^13^C NMR (100 MHz, CDCl_3_) δ 163.9, 150.8, 135.7, 131.1, 129.5, 128.3, 123.8, 77.7, 64.1, 30.7, 30.3, 23.8, 23.3; IR (cm^−1^): 3013, 2940, 2621, 2515, 2099, 1725, 1527, 1272, 1117, 1103; HRESIMS (*m*/*z*): [M^+^ + Na] calcd for C_15_H_16_N_4_O_4_, 339.1069; found, 339.1063.

### Epoxidation of the azidobenzoate **8** with *m*-CPBA


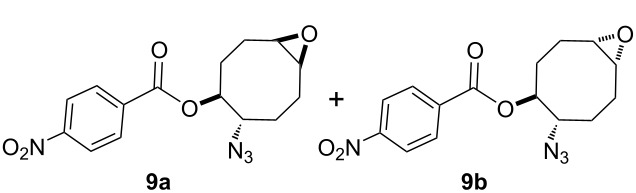


The azidobenzoate **8** (1.00 g, 3.16 mmol) was dissolved in CH_2_Cl_2_ (70 mL) and cooled to 0 °C. *m*-CPBA (77%, 0.73 g, 3.26 mmol) and NaHCO_3_ (0.282 g, 3.36 mmol) were added and the mixture was stirred at room temperature for 36 hours. Then, the solution was cooled to 0 °C and 100 mL of 3 M NaOH added, and stirring continued for 40 min. The mixture was extracted with CH_2_Cl_2_ (4 × 30 mL). The combined organic extracts were washed with saturated aqueous NaCl (20 mL) and then dried (Na_2_SO_4_). Evaporation of solvent gave the mixture of azidobenzoate epoxides **9a** and **9b** (1.02 g, 3.07 mmol, 97%), as light brown solid. The formation and ratio of these isomers was determined by NMR spectroscopy, in a 63:37 ratio (^1^H NMR), but all attempts to separate the isomeric epoxides **9a** and **9b** by column chromatography or crystallization failed.

### Acetylation and methanolysis of the mixture of isomeric azidobenzoate epoxides **9a** and **9b** with HCl(g) in MeOH


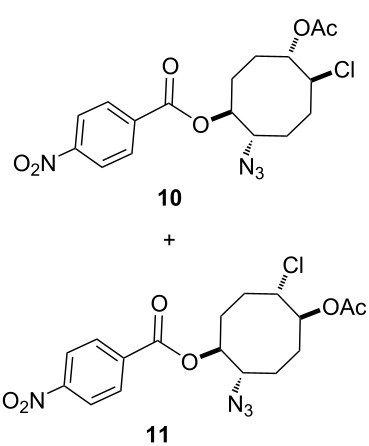


In a similar manner as described in the literature [13], a magnetically stirred solution of the mixture of isomeric epoxides **9a** and **9b** (1.00 g, 3.01 mmol) in 5 mL absolute methanol was cooled to 0 °C. Then, 20 mL absolute methanol containing 10% HCl gas was added and the reaction mixture stirred at 0 °C for 1 hour. The solvent was removed under reduced pressure to obtain the mixture of chlorohydrin-azidobenzoates (1.07 g, 2.90 mmol, 96%). This crude product was dissolved in dichloromethane at 0 °C, and acetyl chloride (0.46 mL, 6.38 mmol) was added dropwise. After 24 hours, evaporation of the solvent under reduced pressure gave the mixture of azidobenzoate chloro-acetates **10** and **11** (1.11 g, 2.70 mmol, total yield 93%). Chromatography of the mixture on a silica gel column eluting with EtOAc/hexane 15:85 gave as the first fraction compound **10** (675 mg, 57%) and as the second fraction compound **11** (417 mg, 35%). Compound **10** was recrystallized from dichloromethane/hexane at room temperature to obtain colourless crystals, mp: 101–102 °C. Compound **11** was recrystallized from the same solvent mixture at 0 °C and obtained as slightly yellow crystals, mp: 117–119 °C. (1S*,2S*,5S*,6S*)-6-Acetoxy-2-azido-5-chlorocyclooctyl 4-nitrobenzoate (**10**): ^1^H NMR (400 MHz, CDCl_3_) δ 8.34–8.22 (m, aromatic, 4H), 5.40–5.33 (m, 1H, H-1), 5.22–5.14 (m, 1H, H-6), 4.25–4.19 (m, 1H, H-5), 3.97 (ddd, *J* = 9.1, 6.0, 3.4 Hz, 1H, H-2), 2.36–1.90 (series of m, 8H, CH_2_), 2.16 (s, 3H, OAc); ^13^C NMR (100 MHz, CDCl_3_) δ 170.2, 164.0, 150.9, 135.3, 131.1, 123.9, 76.9, 75.8, 64.0, 61.5, 26.7, 25.0, 24.5, 21.2; IR: 2951, 2621, 2102, 1772, 1727, 1528, 1272, 1237, 1020, 719; HRESIMS (*m*/*z*): [M^+^ + Na] calcd for C_17_H_19_ClN_4_O_6_, 433.0891; found, 433.0884. (1S*,2S*,5S*,6S*)-5-Acetoxy-2-azido-6-chlorocyclooctyl 4-nitrobenzoate (**11**): ^1^H NMR (400 MHz, CDCl_3_) δ 8.36–8.24 (m, 4H, aromatic), 5.23–5.09 (m, 1H, H-1 and H-5), 4.20 (td, *J* = 8.2, 3.4 Hz, 1H, H-6), 3.91 (td, *J* = 9.3, 2.9 Hz, 1H, H-2), 2.44–1.86 (series of m, 8H, CH_2_), 2.14 (s, 3H, OAc); ^13^C NMR (100 MHz, CDCl_3_) δ 170.0, 163.9, 151.0, 135.2, 131.1, 123.9, 76.9, 75.1, 64.0, 61.4, 29.1, 27.1, 27.0, 25.8, 21.2; IR: 2952, 2515, 2103, 1947, 1728, 1529, 1271, 1239, 1015, 720; HRESIMS (*m*/*z*): [M^+^ + Na] calcd for C_17_H_19_ClN_4_O_6_, 433.0891; found, 433.0885.

## Supporting Information

File 1 Experimental, ^1^H and ^13^C NMR spectra for all new compounds, as well as selected 2D NMR spectra and crystallographic data for compound **10** are provided. Optimized geometries of the transition states with selected interatomic distances and cartesian coordinates for computed structures.

## Data Availability

All data that supports the findings of this study is available in the published article and/or the supporting information of this article.
