# Supplementary material for: Experimental and DFT studies on the regioselective methanolysis of 5-azido-9-oxabicyclo[6.1.0]nonan-4-yl 4-nitrobenzoate isomers
Source: Beilstein J Org Chem. 2026 Mar 26;22:547–56. doi: 10.3762/bjoc.22.40 (PMC13040264; doi:10.3762/bjoc.22.40)
Supplement: File 1 — Experimental, 1H and 13C NMR spectra for all new compounds, as well as selected 2D NMR spectra and crystallographic data for compound 10 are provided. Optimized geometries of the transition states with selected interatomic distances and cartesian coordinates for computed structures. [file Beilstein_J_Org_Chem-22-547-s001.pdf]

BEILSTEIN JOURNAL OF ORGANIC CHEMISTRY

Supporting Information

for

Experimental and DFT studies on the regioselective methanolysis of 5-azido-9-oxabicyclo[6.1.0]nonan-4-yl 4-nitrobenzoate isomers

İlknur Polat, Selçuk Eşsiz and Emine Salamci

Beilstein J. Org. Chem. **2026,** *22,* 547–556. doi:10.3762/bjoc.22.40

Experimental, ^{1}H and ^{13}C NMR spectra for all new compounds, as well as selected 2D NMR spectra and crystallographic data for compound 10 are provided. Optimized geometries of the transition states with selected interatomic distances and cartesian coordinates for computed structures

License and Terms: This is a supporting information file under the terms of the Creative Commons Attribution License (https://creativecommons.org/licenses/by/4.0). Please note that the reuse, redistribution and reproduction in particular requires that the author(s) and source are credited and that individual graphics may be subject to special legal provisions.
The license is subject to the *Beilstein Journal of Organic Chemistry* terms and conditions: (https://www.beilstein-journals.org/bjoc/terms)

Table of contents

1. X-ray crystallographic data S2

2. Copies of NMR spectra S4

3. The optimized geometries and Cartesian coordinates S14

1. X-ray crystallographic data

For the crystal structure determination, a single crystal of the compound **10** was used for data collection on a four-circle Rigaku R-AXIS RAPID-S diffractometer (equipped with a two-dimensional area IP detector). Graphite-monochromated Mo K_α radiation (λ = 0.71073 Å) and oscillation scans technique with Δw = 5º for one image were used for data collection. The lattice parameters were determined by the least-squares methods on the basis of all reflections with $F^2 > 2\sigma(F^2)$. Integration of the intensities, correction for Lorentz and polarization effects and cell refinement were performed using CrystalClear (Rigaku/MSC Inc., 2005) software [1]. The structure was solved by direct methods using SHELXS-97 [2] and non-hydrogen atoms were refined using anisotropic displacement parameters by full-matrix least-squares procedure using the program SHELXL-97 [2]. Hydrogen atoms were positioned geometrically and refined using a riding model. The final difference Fourier maps showed no peaks of chemical significance. Details about the analysed crystal and data collection are presented in Table S1.

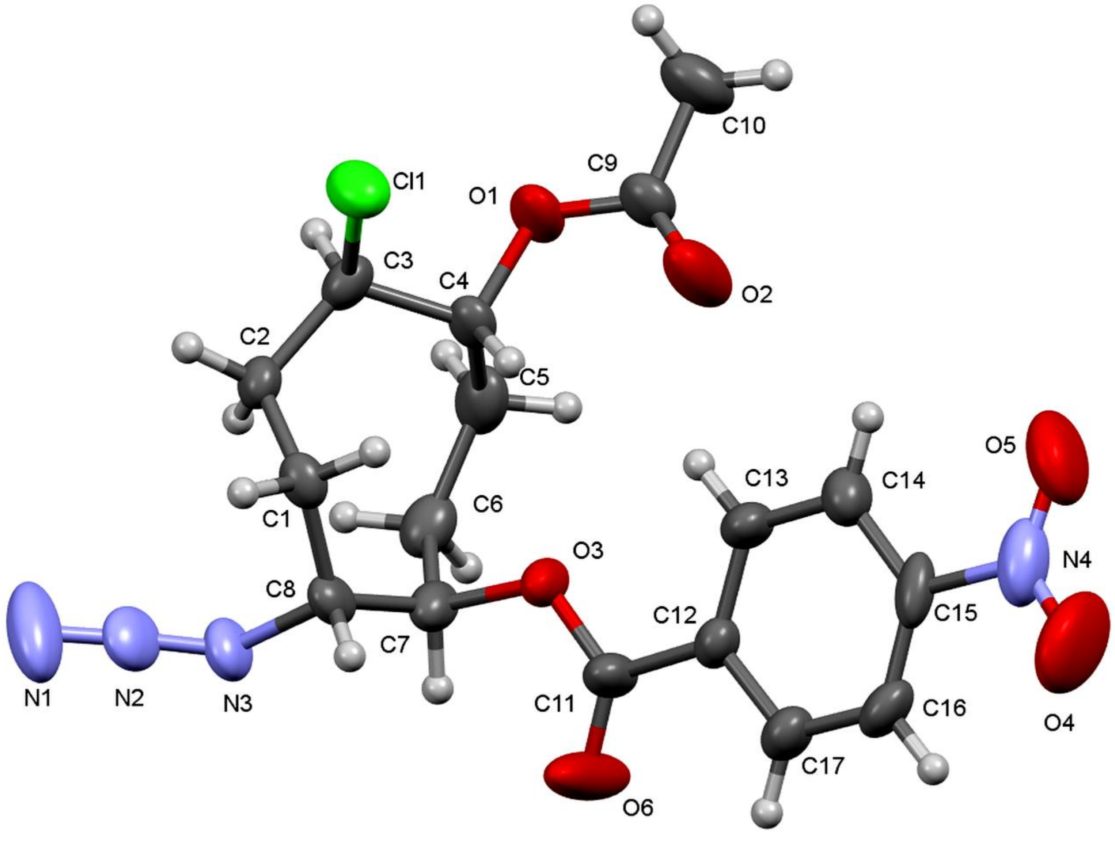

Figure S1. X-ray crystal structure of **10**.

Table S1: Crystal data and structure refinement for compound **10**.

Empirical formula	$C_{17}H_{19}N_4O_6Cl$
Formula weight	410.81
Temperature	293 K
Wavelength	0.71073 A
Crystal system, space group	monoclinic, $P2_1$; (no:4)
Unit cell dimensions	a = 14.1890(8), b = 7.7312(4), c = 18.3459(7) Å, α= 90, β = 108.845(2), γ = 90º
Volume	1904.6(3) $Å^3$
Z, calculated density	4, 1.433 g/cm^3
absorption coefficient	: 0.243 mm^{-1}
$F(000)$	856
θ-range for data collection	2.0-28.4º
refinement method	full matrix least-square on F^2
data/parameters	9349/507
goodness-of-fit on F^2	1.090
final R-indices [$I > 2\sigma(I)$]	R_1 = 0.076, wR_2 = 0.202
largest diff. peak and hole	0.304 and -0.233 e $Å^{-3}$

References

1. Rigaku/MSC, Inc., 9009 new Trails Drive, The Woodlands, TX 77381-5209, USA, 2005.

2. G.M. Sheldrick, SHELXS-97, SHELXL-97 Program for Crystal Structure Solution and refinement, University of Gottingen, Göttingen, Germany, 1997.

2. Copies of NMR spectra

(1S*,8S*,Z)-8-Azidocyclooct-4-en-1-ol (7): $CDCl_3$ (^1H NMR and ^{13}C NMR)

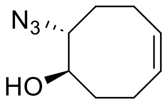

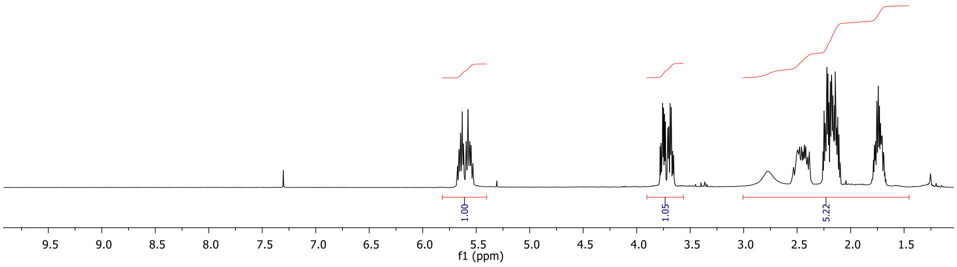

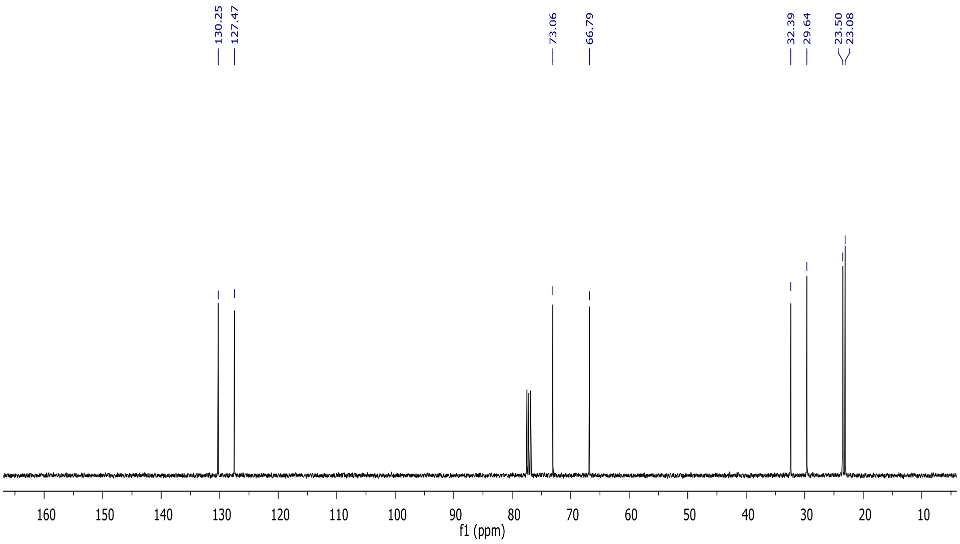

(1*S,8*S**,*Z*)-8-Azidocyclooct-4-en-1-yl 4-nitrobenzoate (8): $CDCl_3$ (^1H NMR and ^{13}C NMR)**

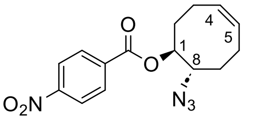

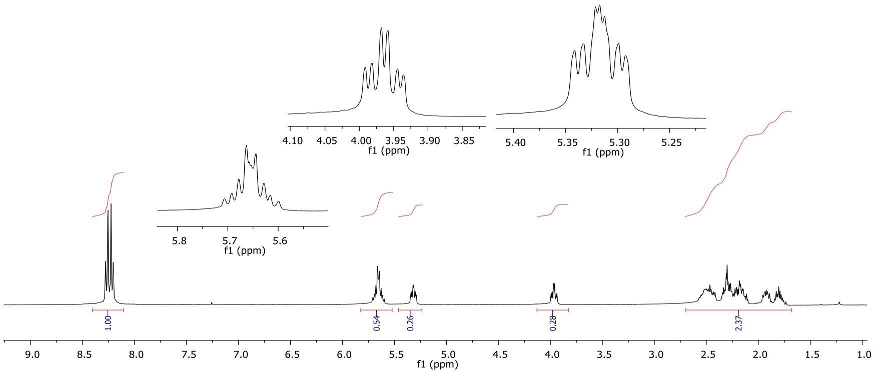

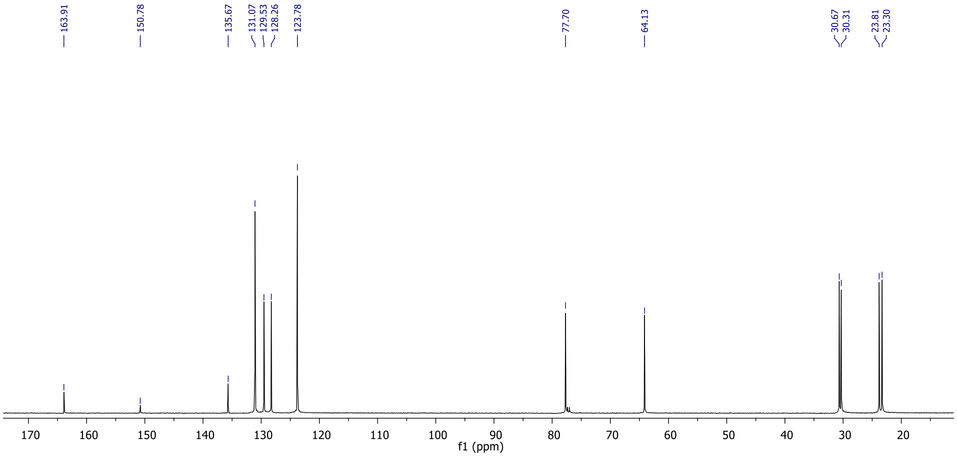

The mixture of isomeric epoxides 9a and 9b: $CDCl_3$ (^1H NMR and ^{13}C NMR)

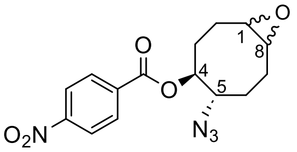

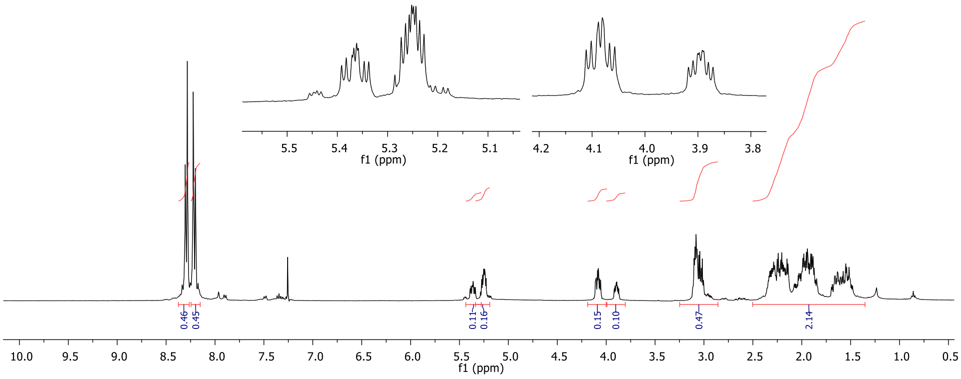

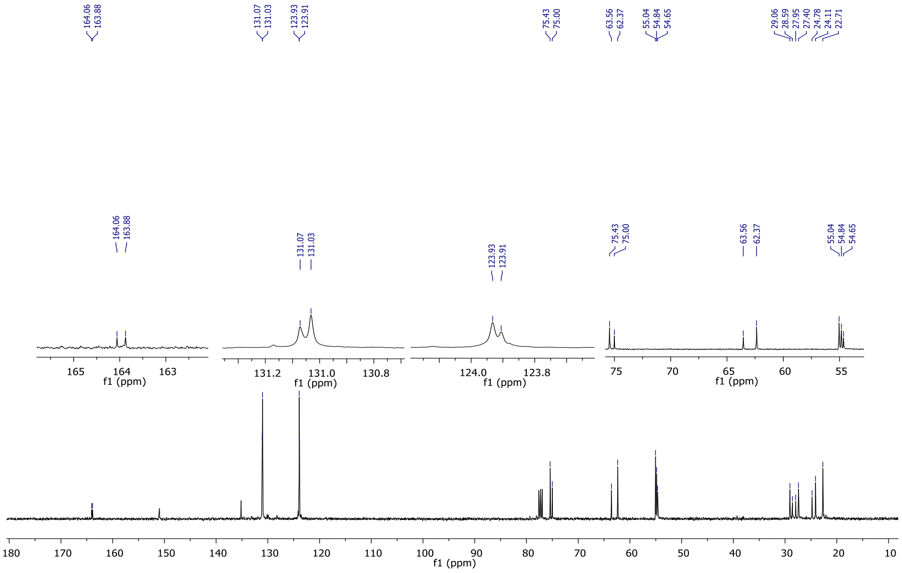

(1*S,2*S**,5*S**,6*S**)-6-Acetoxy-2-azido-5-chlorocyclooctyl 4-nitrobenzoate (10): $CDCl_3$ (^{1}H NMR and ^{13}C NMR)**

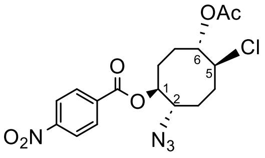

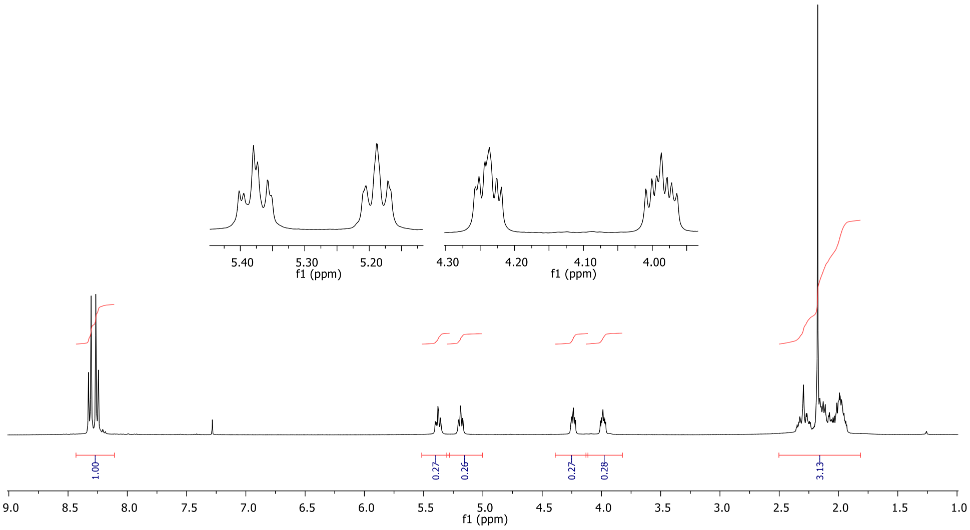

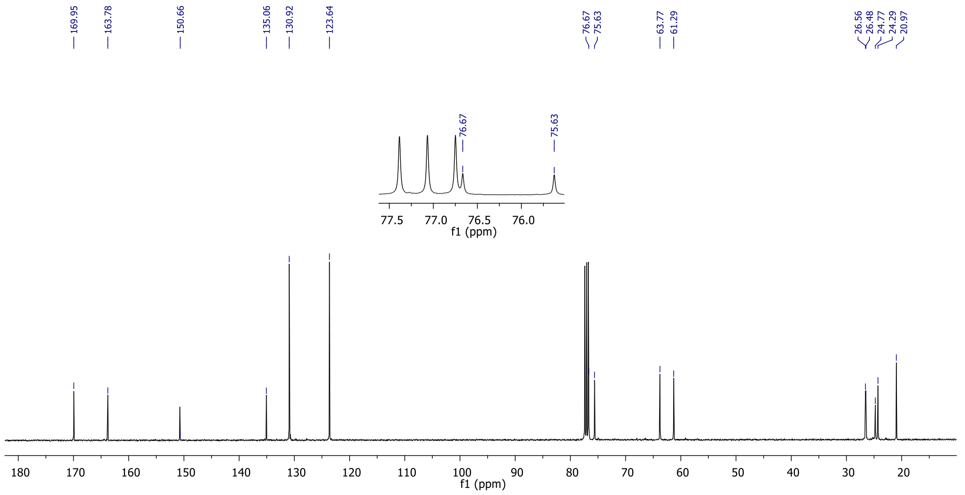

(1*S,2*S**,5*S**,6*S**)-6-Acetoxy-2-azido-5-chlorocyclooctyl 4-nitrobenzoate (10): COSY and HMQC**

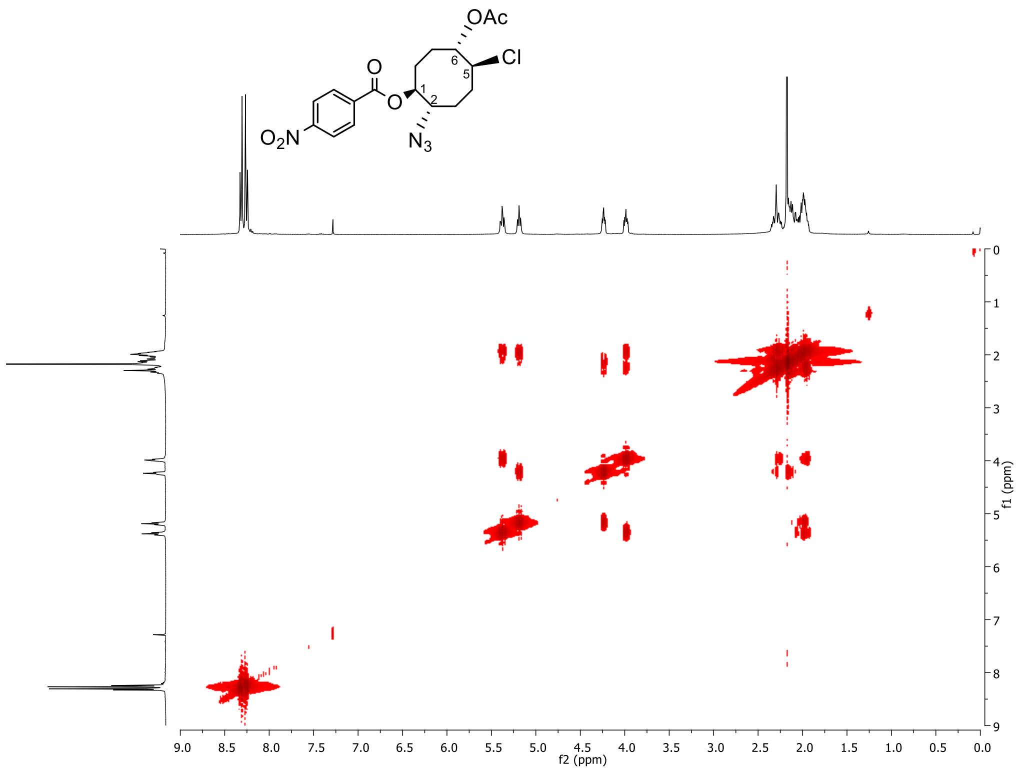

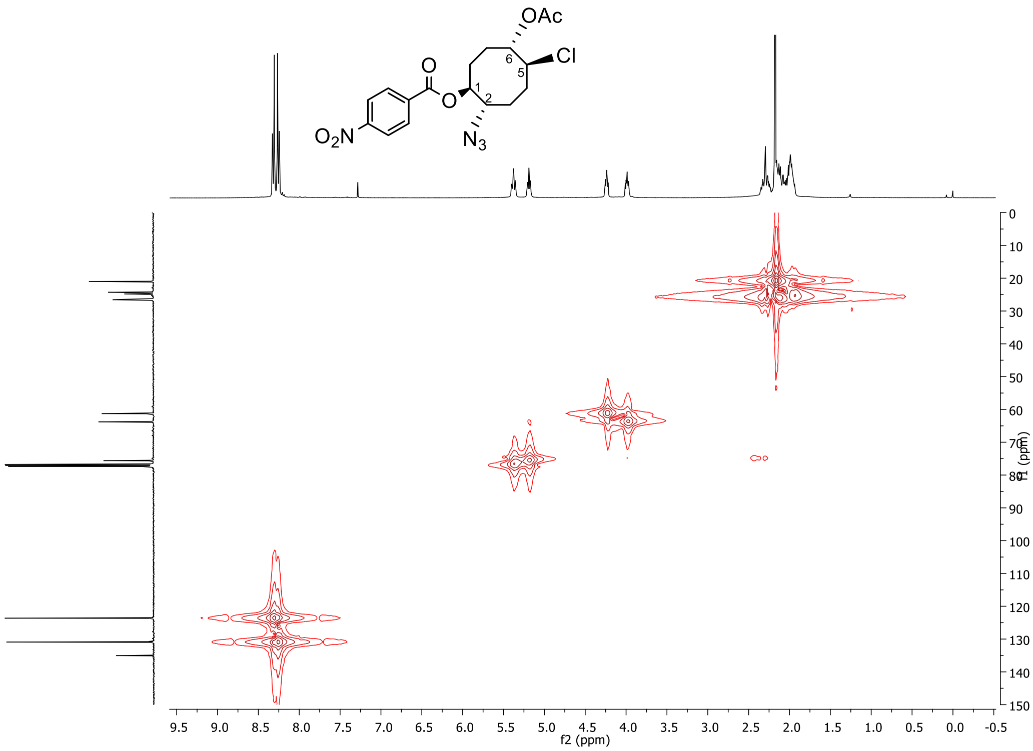

(1*S,2*S**,5*S**,6*S**)-6-Acetoxy-2-azido-5-chlorocyclooctyl 4-nitrobenzoate (10): NOESY**

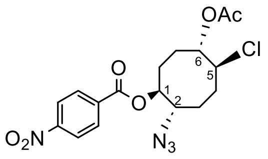

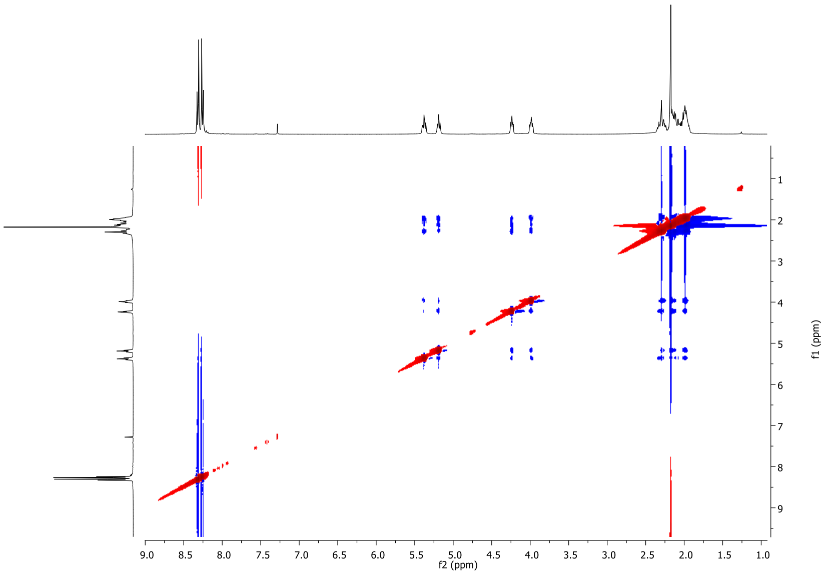

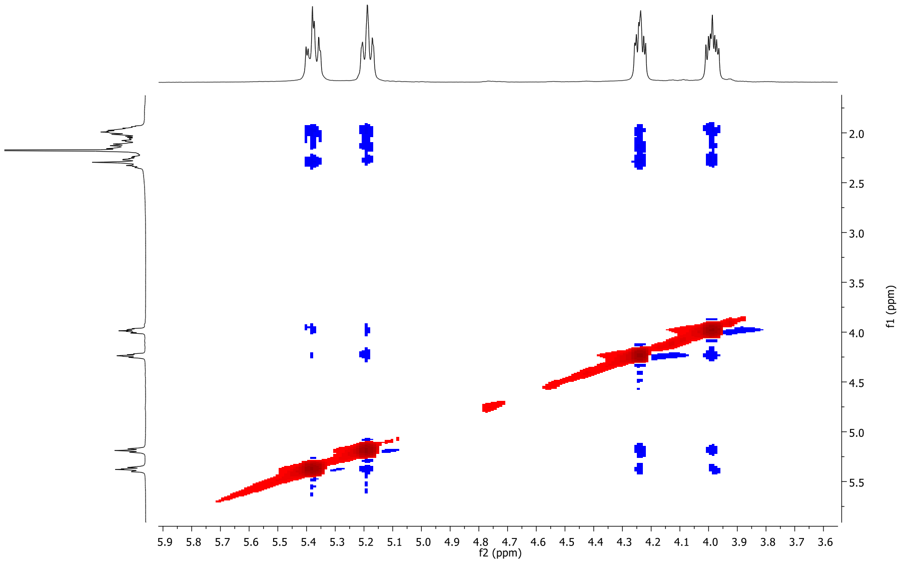

(1*S,2*S**,5*S**,6*S**)-5-Acetoxy-2-azido-6-chlorocyclooctyl 4-nitrobenzoate (11): CDCl3 (^{1}H NMR and ^{13}C NMR)**

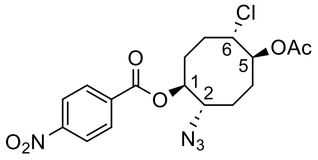

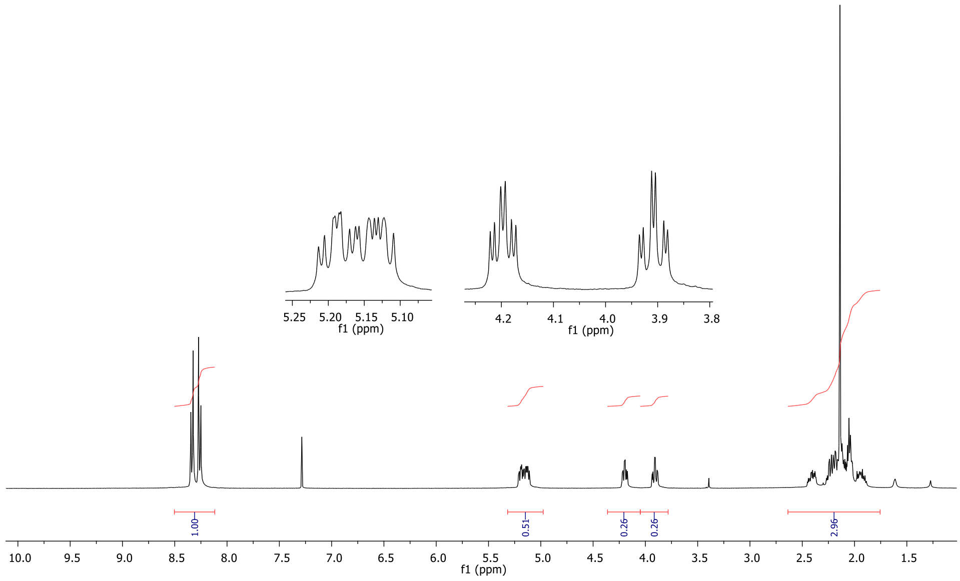

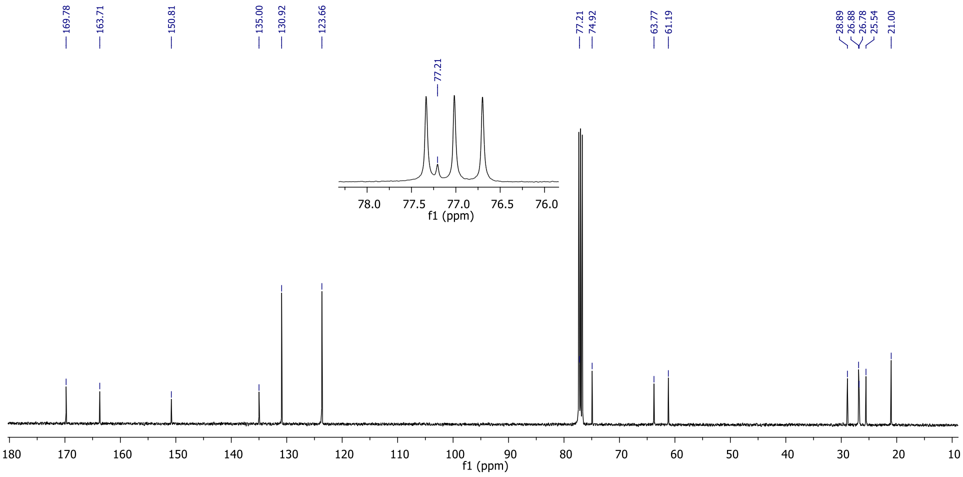

(1*S,2*S**,5*S**,6*S**)-5-Acetoxy-2-azido-6-chlorocyclooctyl 4-nitrobenzoate (11): COSY and HMQC**

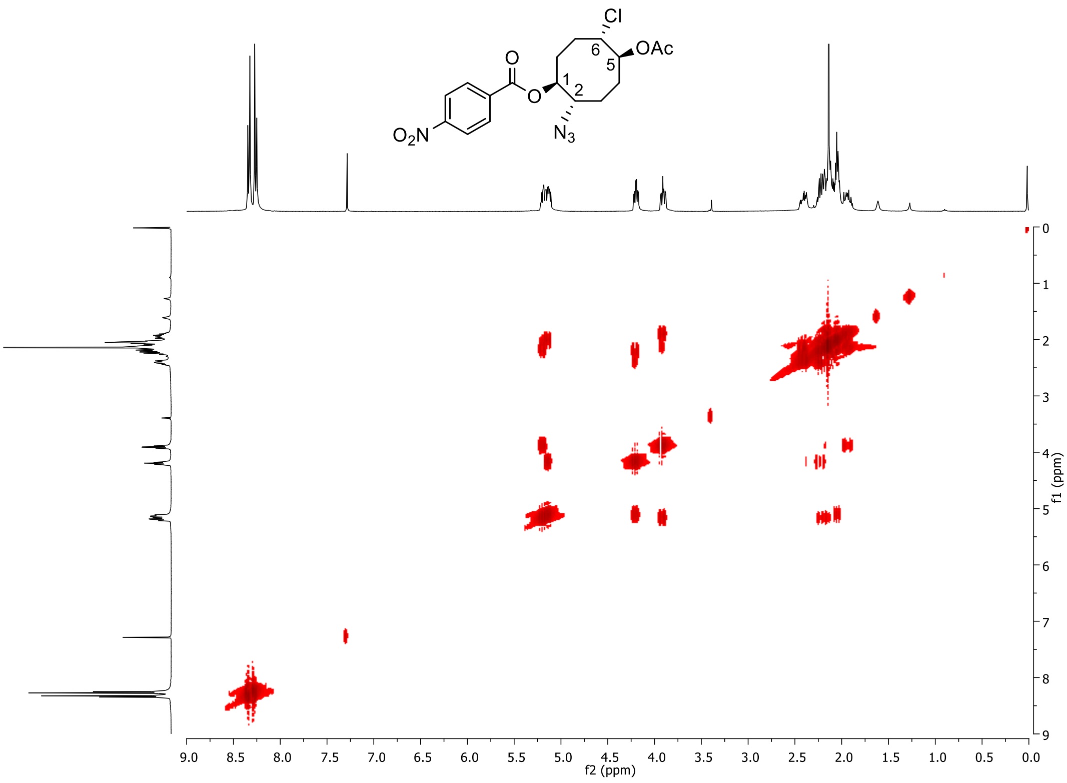

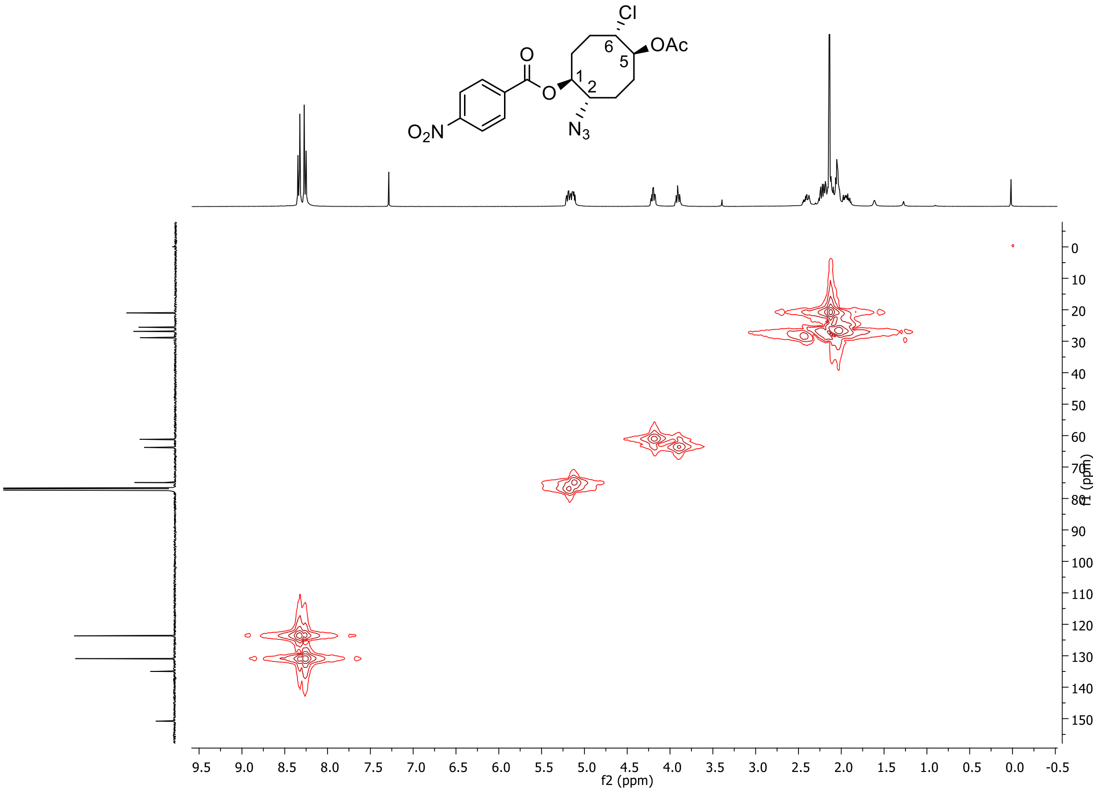

(1*S,2*S**,5*S**,6*S**)-5-Acetoxy-2-azido-6-chlorocyclooctyl 4-nitrobenzoate (11): NOE-Dif spectra**

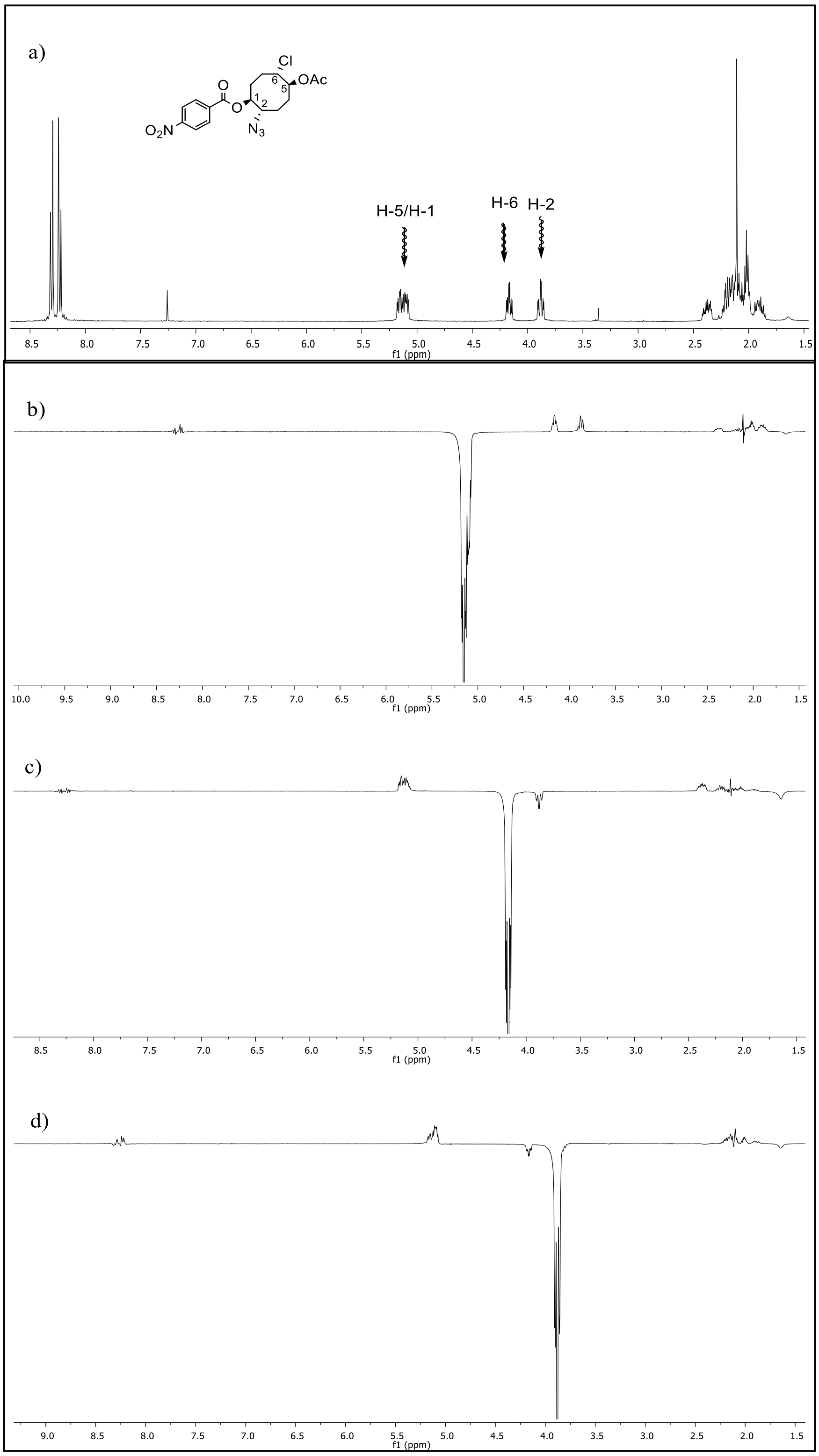

(1*S,2*S**,5*S**,6*S**)-5-Acetoxy-2-azido-6-chlorocyclooctyl 4-nitrobenzoate (11): NOESY**

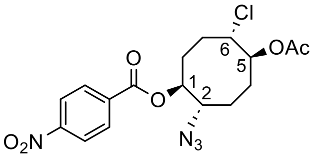

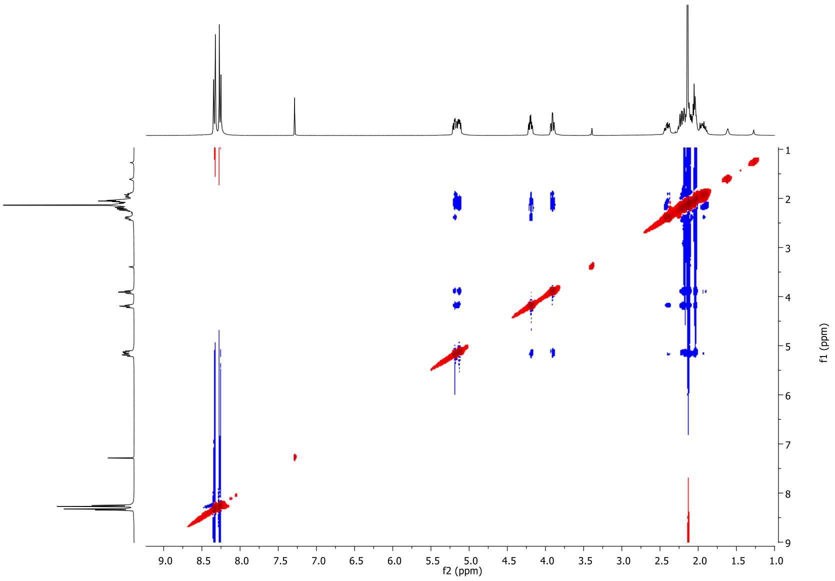

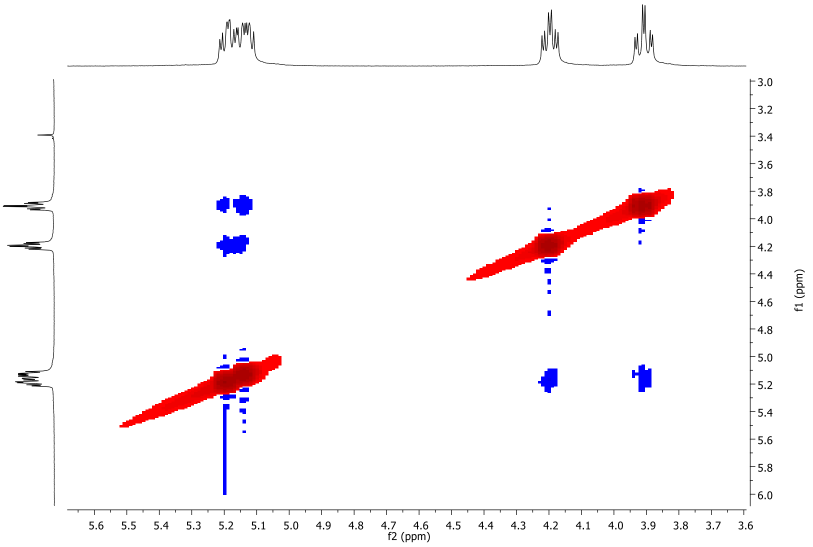

3. The optimized geometries and Cartesian coordinates

9a

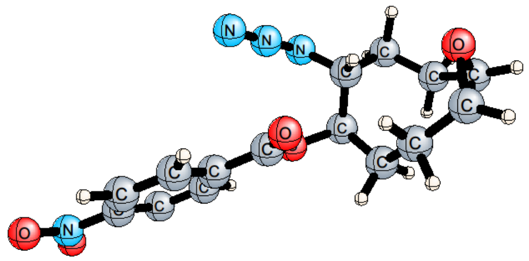

Zero-point correction= 0.313597 (Hartree/Particle)
Thermal correction to Energy= 0.334189
Thermal correction to Enthalpy= 0.335134
Thermal correction to Gibbs Free Energy= 0.260820
Sum of electronic and zero-point Energies= -1175.735927
Sum of electronic and thermal Energies= -1175.715334
Sum of electronic and thermal Enthalpies= -1175.714390
Sum of electronic and thermal Free Energies= -1175.788703
E= -1176.0495234 a.u., number of negative frequencies = 0
0 1

C	-2.32572700	-1.50160900	-0.87536700
C	-1.80404800	-0.05853700	-1.03525100
C	-2.45135000	0.98869500	-0.11338500
C	-3.79906400	1.45387200	-0.66820200
C	-4.77696900	0.39339200	-1.17727600
C	-5.30248400	-0.57819900	-0.13858500
C	-4.51745400	-1.59952600	0.57627000
C	-3.04419900	-1.92214900	0.41713500
O	-0.34873700	-0.02051600	-0.94999700
C	0.26922900	-0.39221900	0.16243900
O	-0.30073900	-0.72467500	1.17610800
C	1.75896400	-0.32666700	0.04114900
O	-4.90082200	-0.37337500	1.22088100
N	-1.61448800	2.22075200	-0.06725000
N	-0.78987800	2.26000800	0.83872800
N	-0.00949100	2.39036300	1.63863100
C	2.51285300	-0.55545500	1.19200400
C	3.89682800	-0.49953200	1.13684900
C	4.48787000	-0.21624800	-0.08606600
C	3.76384900	0.01491000	-1.24632600
C	2.37937800	-0.03895300	-1.17467800
N	5.95910500	-0.15471500	-0.15532700
O	6.47301400	0.04952900	-1.23582000
O	6.58732300	-0.31118000	0.87132200
H	-1.45491300	-2.15174700	-0.99288500
H	-2.97246000	-1.72908000	-1.72464300

H	-1.95430400	0.26601600	-2.06614200
H	-2.55621900	0.59587400	0.89807500
H	-3.57965200	2.12186700	-1.50659500
H	-4.28455000	2.05398200	0.10494100
H	-5.63751200	0.93718000	-1.57419100
H	-4.35902900	-0.15393800	-2.02544200
H	-6.36404400	-0.79430700	-0.23168400
H	-5.09838500	-2.43743900	0.95403400
H	-2.98055300	-3.01276400	0.44999600
H	-2.51242200	-1.56825700	1.30216100
H	2.01420300	-0.77446000	2.12796200
H	4.50088300	-0.67165900	2.01727500
H	4.26786600	0.23436800	-2.17778600
H	1.78798100	0.14058900	-2.06276900

12

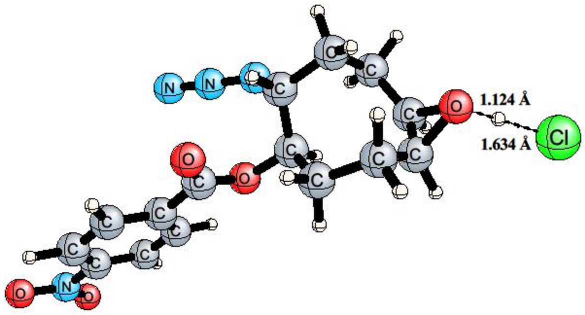

Zero-point correction= 0.323055 (Hartree/Particle)
Thermal correction to Energy= 0.346016
Thermal correction to Enthalpy= 0.346960
Thermal correction to Gibbs Free Energy= 0.264052
Sum of electronic and zero-point Energies= -1636.548808
Sum of electronic and thermal Energies= -1636.525848
Sum of electronic and thermal Enthalpies= -1636.524903
Sum of electronic and thermal Free Energies= -1636.607812
E= -1636.8718634 a.u., number of negative frequencies = 0
0 1

C	-1.61388200	-0.61398700	1.40108600
C	-1.14641000	0.16576500	0.17327800
C	-1.46019100	1.66988600	0.12674700
C	-2.95030700	2.00516300	-0.00548500
C	-3.69405600	1.22712000	-1.09068000
C	-4.15409000	-0.17534000	-0.79831600
C	-3.90398500	-1.02197600	0.38167800
C	-3.13045300	-0.64261300	1.61506000
O	0.27658200	-0.06969800	-0.02848600
C	1.17975100	0.33289100	0.86259700
O	0.92573400	0.97510900	1.85400700
C	2.56849100	-0.07261800	0.48030300
O	-5.19335500	-0.32183500	0.24288700

N	-0.80780800	2.23710100	-1.09283300
C	2.82404900	-0.78243100	-0.69314100
C	4.12687100	-1.13208200	-1.01722200
C	5.14034300	-0.75378200	-0.14934400
C	4.91501300	-0.04544300	1.02203800
C	3.60811200	0.29562800	1.33377100
N	0.36534300	2.55716200	-0.94741300
N	1.44048200	2.88608900	-0.89974500
N	6.52688100	-1.12082700	-0.48955100
O	7.41820900	-0.71462600	0.22700500
O	6.71145700	-1.81211900	-1.47000900
H	-1.15366200	-0.21037600	2.30283800
H	-1.24628600	-1.63588300	1.27081100
H	-1.55637300	-0.28995900	-0.72988200
H	-1.06108900	2.16867500	1.01217900
H	-2.99408300	3.06467300	-0.26478100
H	-3.44754200	1.91698100	0.95849400
H	-3.07215400	1.15894100	-1.98953600
H	-4.58805800	1.78140500	-1.39015500
H	-4.44936500	-0.73081300	-1.68451400
H	-4.02202800	-2.08701900	0.20091900
H	-3.35013100	-1.38495300	2.38457400
H	-3.48954600	0.31162900	1.99265400
H	2.01367300	-1.06098700	-1.35353900
H	4.35019000	-1.68131900	-1.92181200
H	5.73662000	0.22858000	1.66972400
H	3.39131300	0.84784700	2.23960200
H	-6.02101300	-1.01071100	-0.08060400
Cl	-7.21643000	-2.02912400	-0.53339000

14

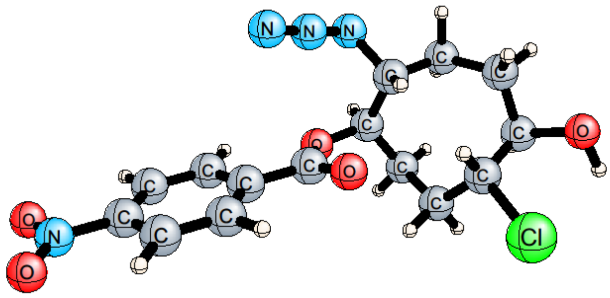

Zero-point correction= 0.327653 (Hartree/Particle)
Thermal correction to Energy= 0.350272
Thermal correction to Enthalpy= 0.351217
Thermal correction to Gibbs Free Energy= 0.272677
Sum of electronic and zero-point Energies= -1636.573017
Sum of electronic and thermal Energies= -1636.550398
Sum of electronic and thermal Enthalpies= -1636.549453
Sum of electronic and thermal Free Energies= -1636.627993

E= -1636.90067 a.u., number of negative frequencies = 0
0 1

C	2.08627300	0.11616700	1.90388100
C	1.28776800	1.17051800	1.14796800
C	1.86831300	1.71155400	-0.16102800
C	3.33959100	2.10539200	0.00595500
C	4.35180700	1.10180000	-0.55214800
C	4.54737100	-0.27410400	0.09624400
C	3.28245300	-1.16745500	-0.01703800
C	2.44305000	-1.22256200	1.25654100
O	-0.11887500	0.81327500	1.00745200
O	5.66862200	-0.80137400	-0.60244100
Cl	3.72463500	-2.90400500	-0.38915400
N	1.09923000	2.94224300	-0.52502400
N	0.03444800	2.72210000	-1.09259400
N	-0.94943300	2.61330700	-1.62801700
C	-0.54320400	-0.06990600	0.10836400
C	-2.03438500	-0.20361500	0.11079100
O	0.17260400	-0.68319300	-0.64723500
C	-2.83948200	0.62421600	0.89379800
C	-4.21942700	0.48861100	0.84738500
C	-4.75317500	-0.48275500	0.01353200
C	-3.97650100	-1.32087600	-0.77341300
C	-2.59956800	-1.16989900	-0.72095100
N	-6.21885200	-0.63015700	-0.04154800
O	-6.67933600	-1.51472100	-0.73341200
O	-6.89614400	0.14008400	0.60746400
H	1.51417100	-0.10440400	2.80939300
H	2.99629600	0.61282500	2.24954000
H	1.21973200	2.03096100	1.81740800
H	1.76333100	0.99999200	-0.98010300
H	3.55520400	2.34653300	1.05072200
H	3.48708300	3.02818400	-0.55797300
H	4.12451500	0.92260900	-1.60939000
H	5.33280900	1.58234700	-0.51583100
H	4.80658100	-0.17328700	1.15710300
H	2.69136600	-0.88047400	-0.88397100
H	3.02213000	-1.77497400	2.00315400
H	1.54140400	-1.80184800	1.05685500
H	5.90361100	-1.65739200	-0.22609800
H	-2.39519200	1.37453200	1.53434600
H	-4.86398500	1.12089000	1.44277900
H	-4.43535100	-2.06447500	-1.41071800
H	-1.96050700	-1.80147600	-1.32522600

13

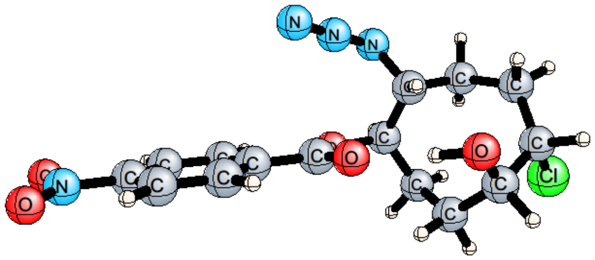

Zero-point correction= 0.328081 (Hartree/Particle)
Thermal correction to Energy= 0.350547
Thermal correction to Enthalpy= 0.351491
Thermal correction to Gibbs Free Energy= 0.272762
Sum of electronic and zero-point Energies= -1636.572090
Sum of electronic and thermal Energies= -1636.549624
Sum of electronic and thermal Enthalpies= -1636.548680
Sum of electronic and thermal Free Energies= -1636.627409
E= -1636.9001706 a.u., number of negative frequencies = 0
0 1

C	2.12240100	-0.67136300	1.50816300
C	1.37291000	0.58593100	1.08432500
C	1.92911100	1.39859200	-0.08293700
C	3.42465500	1.68399300	0.10667000
C	4.34025700	0.86904600	-0.80735500
C	4.55255000	-0.62784500	-0.59590700
C	3.35587900	-1.59864200	-0.68461400
C	2.46991800	-1.81558700	0.55064700
O	-0.06749200	0.37385800	0.95419200
Cl	5.47819300	-0.95184400	0.94200200
O	2.62542400	-1.22167000	-1.85051400
N	1.20390100	2.70658800	-0.12108400
N	0.12413200	2.66436900	-0.70092500
N	-0.87077500	2.71655800	-1.22449200
C	-0.61950500	-0.26363000	-0.06439800
C	-2.11273300	-0.29176800	0.02579700
O	-0.01317200	-0.75861400	-0.98984700
C	-2.78914600	0.35345400	1.06148700
C	-4.17524800	0.32010300	1.10546300
C	-4.84444200	-0.36661400	0.10340900
C	-4.19751400	-1.01687600	-0.93734600
C	-2.81240700	-0.97074900	-0.97140700
N	-6.31758500	-0.40748200	0.14676700
O	-6.89497800	-1.09004000	-0.67400900
O	-6.88281200	0.24323400	1.00118500
H	1.53093700	-1.10290200	2.32154200
H	3.04153100	-0.31870300	1.97986800
H	1.39812800	1.25153000	1.94989500
H	1.77039800	0.89952300	-1.03839000

H	3.71372900	1.58190500	1.15677200
H	3.58508200	2.73205400	-0.15235700
H	3.98781300	0.97718100	-1.83788100
H	5.33470800	1.32170300	-0.77691800
H	5.25203600	-0.93835600	-1.37215900
H	3.79549200	-2.57952500	-0.89448700
H	1.56376400	-2.31796400	0.20122100
H	2.98886900	-2.55122700	1.17043400
H	1.68930100	-1.12937700	-1.62151000
H	-2.24011200	0.88048300	1.83054100
H	-4.72161500	0.81272600	1.89822600
H	-4.76029400	-1.53825500	-1.69967600
H	-2.27265200	-1.46208000	-1.77104800

TS1

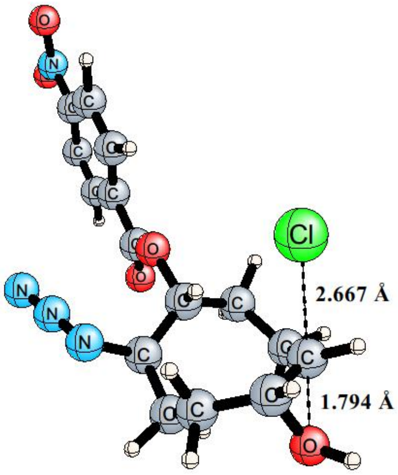

Zero-point correction= 0.325720 (Hartree/Particle)
Thermal correction to Energy= 0.348523
Thermal correction to Enthalpy= 0.349467
Thermal correction to Gibbs Free Energy= 0.269643
Sum of electronic and zero-point Energies= -1636.521274
Sum of electronic and thermal Energies= -1636.498471
Sum of electronic and thermal Enthalpies= -1636.497527
Sum of electronic and thermal Free Energies= -1636.577351
E= -1636.8469939 a.u., im. freq.= 412.44*i* cm^{-1}
0 1

C	-1.98408400	-0.49350900	1.48242800
C	-1.56847800	0.16305000	0.17102500
C	-1.99843400	1.61469300	-0.04928400
C	-3.51100800	1.76237900	-0.21521600
C	-4.14465900	0.78191000	-1.20937000
C	-4.71515200	-0.50653000	-0.67156500
C	-3.48438900	-0.77674900	1.66986200
O	-0.13086000	0.01096300	-0.00554800
C	0.73200100	0.56658400	0.83943600

O	0.42500300	1.30270600	1.74781000
C	2.14759400	0.20231800	0.51885900
O	-5.73698600	-0.25854400	0.36025800
N	-1.39188600	2.09814400	-1.32684700
C	2.45172700	-0.73098500	-0.47244800
C	3.77654500	-1.04089600	-0.74366100
C	4.76176600	-0.39976500	-0.00758000
C	4.48763300	0.53197300	0.98321500
C	3.15946400	0.83188700	1.24320400
N	-0.24220900	2.50746700	-1.22349400
N	0.80870600	2.90993000	-1.21558400
N	6.17104500	-0.72561900	-0.29135300
O	7.02583300	-0.22941700	0.41331100
O	6.41048300	-1.47470000	-1.21584200
H	-1.66369700	0.11094600	2.33167700
H	-1.45032900	-1.44423800	1.51936400
H	-1.93916700	-0.43882100	-0.65778500
H	-1.65975600	2.25087500	0.77102200
H	-3.68029200	2.77822600	-0.57575300
H	-3.99624100	1.71067700	0.75939700
H	-3.44040900	0.52105200	-2.00538800
H	-4.98827500	1.26745800	-1.70716400
H	-5.11785800	-1.13239000	-1.46265200
H	-3.58175200	-1.51507700	2.46614700
H	-3.98955500	0.12457100	2.02028100
H	-6.50617400	-0.83419500	0.21593000
H	1.66141600	-1.21802800	-1.02824600
H	4.03722100	-1.76310200	-1.50530800
H	5.28863500	1.00967200	1.53077700
H	2.90485700	1.55584500	2.00708900
C	-4.26841500	-1.27956300	0.49444300
H	-4.72339700	-2.25572300	0.56277000
Cl	-2.56325200	-2.96623800	-0.67301200

TS2

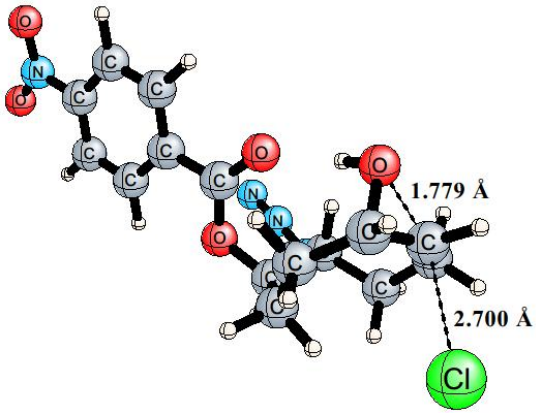

Zero-point correction= 0.325977 (Hartree/Particle)

Thermal correction to Energy= 0.348662
Thermal correction to Enthalpy= 0.349606
Thermal correction to Gibbs Free Energy= 0.269905
Sum of electronic and zero-point Energies= -1636.513752
Sum of electronic and thermal Energies= -1636.491067
Sum of electronic and thermal Enthalpies= -1636.490123
Sum of electronic and thermal Free Energies= -1636.569824
E= -1636.8397284 a.u., im. freq.= 394.68*i* cm^{-1}
0 1

C	-2.17315900	-0.35803700	-1.60470300
C	-1.36587200	0.79789500	-1.02320000
C	-1.83136300	1.51250400	0.24967100
C	-3.34951000	1.66153700	0.29185600
C	-4.04615300	0.65267400	1.19860200
C	-4.03999400	-0.83226200	1.00738900
C	-3.21374400	-1.82895700	0.32570000
C	-2.35369300	-1.70306800	-0.90880500
O	0.06103700	0.50121700	-0.94707300
Cl	-5.88461200	-0.89884000	-0.96286300
O	-2.57449000	-1.61991600	1.63821600
N	-1.22019600	2.87488200	0.26545400
N	-0.03875200	2.89251900	0.59983500
N	1.03748900	3.00807000	0.90539300
C	0.57972200	-0.23859100	0.01931500
C	2.07081400	-0.30066500	-0.04978100
O	-0.05735300	-0.79982300	0.88741000
C	2.78451700	0.47493700	-0.96418400
C	4.17001300	0.41073600	-0.98813700
C	4.79995300	-0.43888300	-0.09121600
C	4.11533600	-1.22124600	0.82745800
C	2.73148300	-1.14201100	0.84507000
N	6.27264300	-0.51266100	-0.11444100
O	6.81650200	-1.31251500	0.61845800
O	6.87004700	0.23005200	-0.86538400
H	-1.69570800	-0.57398800	-2.56471700
H	-3.16133900	0.03710500	-1.85078100
H	-1.38220500	1.56367700	-1.80166400
H	-1.50056600	0.99765800	1.15222000
H	-3.78302300	1.65609300	-0.70981000
H	-3.57163300	2.63971300	0.72197200
H	-3.70034400	0.79250400	2.23089100
H	-5.11143100	0.89347800	1.22437700
H	-4.77641800	-1.30960500	1.64366100
H	-3.67468700	-2.81083400	0.36733500
H	-1.39752300	-2.18437000	-0.68636000
H	-2.84108200	-2.34611300	-1.64564500
H	-1.74193400	-1.11728700	1.50706000
H	2.26548600	1.12879100	-1.65258900
H	4.74548600	1.00350000	-1.68604500

H	4.64886800	-1.86808000	1.51060300
H	2.16213600	-1.73313300	1.55124300

9b

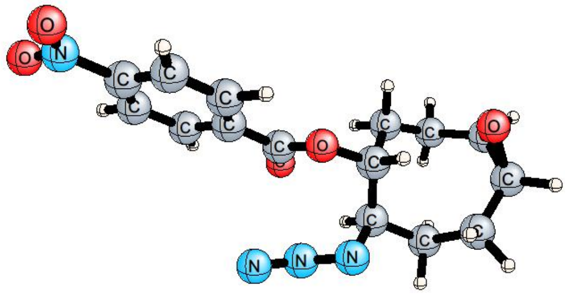

Zero-point correction= 0.313484 (Hartree/Particle)
Thermal correction to Energy= 0.334200
Thermal correction to Enthalpy= 0.335144
Thermal correction to Gibbs Free Energy= 0.260377
Sum of electronic and zero-point Energies= -1175.736409
Sum of electronic and thermal Energies= -1175.715694
Sum of electronic and thermal Enthalpies= -1175.714750
Sum of electronic and thermal Free Energies= -1175.789517
E= -1176.0498936 a.u., number of negative frequencies = 0
0 1

C	2.31682600	-1.27817700	-1.05341500
C	1.92564000	-0.22822100	-0.01524100
C	2.42709400	1.20318100	-0.21835000
C	3.94261500	1.33969200	-0.06682700
C	4.58257500	0.60138100	1.11684600
C	5.04976000	-0.82996400	0.91490500
C	4.72795600	-1.75005600	-0.19197200
C	3.80364200	-1.48618400	-1.36678200
O	0.47359000	-0.25984000	0.14565700
C	-0.34585700	0.11509400	-0.83038900
O	0.00955000	0.58567000	-1.88617700
C	-1.78503200	-0.07658900	-0.46416900
O	4.13827100	-1.92549800	1.10172300
N	1.82189300	2.05767700	0.85157100
N	0.70956000	2.49676100	0.59016900
N	-0.30823800	2.94929000	0.42606500
C	-2.15799800	-0.67045200	0.74185200
C	-3.50240700	-0.82387000	1.04830000
C	-4.43819800	-0.37251800	0.12949900
C	-4.09550900	0.22290700	-1.07601800
C	-2.74846600	0.36896000	-1.36877200
N	-5.86802400	-0.53434400	0.44928800
O	-6.68293000	-0.17764200	-0.37652700
O	-6.16328900	-1.01717700	1.52302800
H	1.81400900	-1.05508400	-1.99588400

H	1.90771900	-2.22264000	-0.68318600
H	2.25029200	-0.55113400	0.97161400
H	2.12816100	1.58994900	-1.19424500
H	4.13131700	2.40839800	0.05444100
H	4.43094400	1.05550900	-0.99774100
H	5.48156300	1.15794000	1.39105900
H	3.93303200	0.64019900	1.99867900
H	5.99961000	-1.03011400	1.40501300
H	5.49432200	-2.49213300	-0.40331300
H	3.84791200	-2.36982500	-2.00828700
H	4.19941200	-0.66879800	-1.97089200
H	-1.40615200	-1.01384900	1.43990000
H	-3.81573400	-1.28345900	1.97586200
H	-4.85884500	0.56317600	-1.76243400
H	-2.44127700	0.83095800	-2.29866600

15

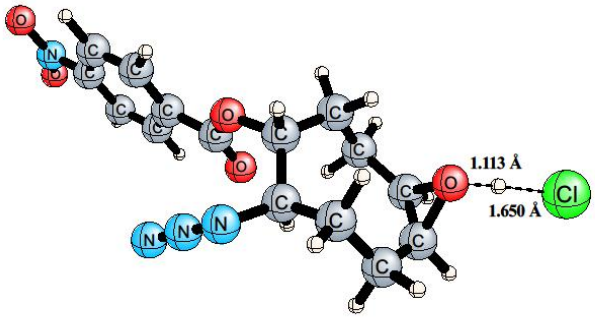

Zero-point correction= 0.323171 (Hartree/Particle)
Thermal correction to Energy= 0.346231
Thermal correction to Enthalpy= 0.347175
Thermal correction to Gibbs Free Energy= 0.265032
Sum of electronic and zero-point Energies= -1636.542562
Sum of electronic and thermal Energies= -1636.519502
Sum of electronic and thermal Enthalpies= -1636.518558
Sum of electronic and thermal Free Energies= -1636.600700
E= -1636.8657327 a.u., number of negative frequencies = 0
0 1

C	1.79249100	-0.88975200	1.07701900
C	1.00999900	0.42746600	1.11548900
C	1.56598800	1.50485900	0.19093800
C	2.96789300	1.89706100	0.68100900
C	4.05442500	1.72848100	-0.36835000
C	4.47193600	0.33259400	-0.72398300
C	3.77442700	-0.96339600	-0.66059300
C	2.38597800	-1.38694600	-0.24838600
O	-0.42573500	0.23304400	0.95783300
C	-0.97324900	-0.17267800	-0.18069400
O	-0.36030700	-0.37722600	-1.20226700
C	-2.45889200	-0.31952400	-0.07347200

O	4.72996500	-0.59211900	0.40501200
N	0.70395300	2.71784000	0.23514600
C	-3.13061800	-0.07527200	1.12452200
C	-4.50981500	-0.21372600	1.18243500
C	-5.17759300	-0.59518000	0.02825500
C	-4.53528200	-0.84334700	-1.17623300
C	-3.15711100	-0.70055200	-1.21916200
N	-0.25615500	2.69194900	-0.52892600
N	-1.15397200	2.76111700	-1.20287400
N	-6.64326100	-0.74344300	0.08363800
O	-7.22197800	-1.08542000	-0.92687600
O	-7.20198500	-0.51683600	1.13702300
H	2.59097600	-0.82269100	1.81920900
H	1.11146900	-1.65969100	1.44691400
H	1.04724200	0.81626900	2.13399500
H	1.60507100	1.14362900	-0.83987400
H	3.23413800	1.33580500	1.58028500
H	2.93826900	2.94702700	0.97469100
H	3.75613300	2.20583000	-1.30801400
H	4.96251000	2.24039800	-0.03733000
H	5.31603200	0.31202300	-1.40938800
H	4.23437600	-1.69352500	-1.32215600
H	1.73401900	-1.17329900	-1.09672800
H	2.43955700	-2.47591800	-0.17786500
H	-2.58320800	0.22141800	2.00931300
H	-5.05240600	-0.02959900	2.09973200
H	-5.09674100	-1.13740600	-2.05260600
H	-2.62048400	-0.88408500	-2.14157400
H	5.70698300	-1.11541800	0.29912700
Cl	7.14582100	-1.87618200	0.02675400

16

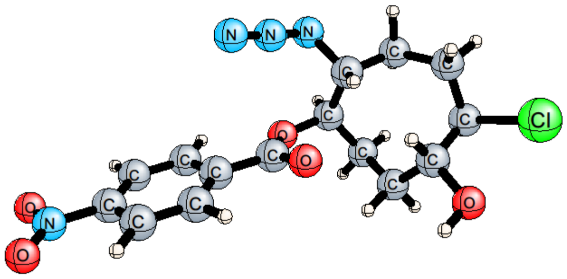

Zero-point correction= 0.327268 (Hartree/Particle)
Thermal correction to Energy= 0.350028
Thermal correction to Enthalpy= 0.350972
Thermal correction to Gibbs Free Energy= 0.271732
Sum of electronic and zero-point Energies= -1636.570728
Sum of electronic and thermal Energies= -1636.547967
Sum of electronic and thermal Enthalpies= -1636.547023
Sum of electronic and thermal Free Energies= -1636.626264

E= -1636.8979956 a.u., number of negative frequencies = 0
0 1

C	1.95398600	-0.07258400	1.92628100
C	1.18422900	1.01894600	1.19274800
C	1.79042100	1.57746500	-0.09655100
C	3.26133100	1.95660900	0.10465400
C	4.28642100	0.96999000	-0.46940500
C	4.42748400	-0.41872200	0.16301800
C	3.19443500	-1.33207800	-0.03041800
C	2.32356000	-1.38098800	1.23083500
O	-0.22852800	0.69499600	1.02946500
Cl	5.91849300	-1.14578900	-0.59673800
O	3.60124900	-2.66695400	-0.32186200
N	1.04001300	2.82191300	-0.45235600
N	-0.01282500	2.62311100	-1.04913600
N	-0.98480100	2.53400900	-1.60941400
C	-0.66541500	-0.15476000	0.10533900
O	0.04091100	-0.76185900	-0.66417300
O	-6.82471000	-1.45215300	-0.80606400
C	-2.15916600	-0.25835300	0.09735000
C	-2.95244300	0.57805500	0.88328600
C	-4.33453400	0.47157500	0.82601200
C	-4.88243000	-0.48033800	-0.02100000
C	-4.11769800	-1.32662400	-0.81092900
C	-2.73837100	-1.20439800	-0.74790600
N	-6.35041000	-0.59664500	-0.08746300
O	-7.01578100	0.16880400	0.57928100
H	1.35789900	-0.32058700	2.80896100
H	2.85768500	0.40436600	2.31495000
H	1.12585200	1.86395300	1.88249400
H	1.69345000	0.87973000	-0.92792900
H	3.46404000	2.16366500	1.15919000
H	3.42430700	2.89489600	-0.42874500
H	4.08013000	0.82911200	-1.53575700
H	5.26030200	1.45733000	-0.39271600
H	4.68959900	-0.35025300	1.21940800
H	2.62480000	-0.95843300	-0.88619500
H	2.88903200	-1.97144500	1.96009000
H	1.42290000	-1.95073000	0.99046200
H	2.79878500	-3.19232300	-0.42834100
H	-2.49725200	1.31296300	1.53397700
H	-4.97016300	1.11157500	1.42274300
H	-4.58734700	-2.05458400	-1.45834400
H	-2.10822100	-1.84298400	-1.35416000

17

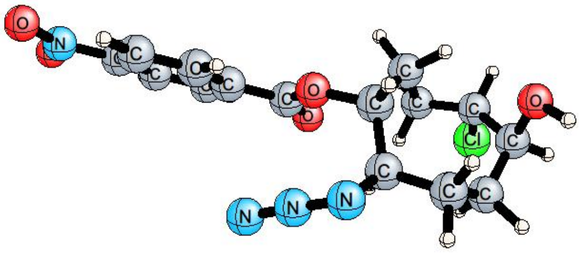

Zero-point correction= 0.327355 (Hartree/Particle)
Thermal correction to Energy= 0.350129
Thermal correction to Enthalpy= 0.351073
Thermal correction to Gibbs Free Energy= 0.271602
Sum of electronic and zero-point Energies= -1636.573240
Sum of electronic and thermal Energies= -1636.550467
Sum of electronic and thermal Enthalpies= -1636.549522
Sum of electronic and thermal Free Energies= -1636.628993
E= -1636.9005952 a.u., number of negative frequencies = 0
0 1

C	1.95607000	-0.50010100	1.35312300
C	1.24416000	0.77811000	0.89001000
C	1.74576000	1.44872900	-0.39368500
C	3.18950500	1.95266500	-0.29008200
C	4.33722800	1.00643100	-0.67650900
C	4.88179100	0.04036000	0.38723700
C	4.18576300	-1.32654100	0.46052400
C	2.67653100	-1.34896800	0.28870200
O	-0.20355100	0.59813800	0.80777300
O	4.82939500	0.59381300	1.70140800
Cl	4.90023200	-2.37738100	-0.85039600
N	0.90653000	2.66802000	-0.61839600
N	-0.15337100	2.45672900	-1.19515600
N	-1.13856000	2.35452900	-1.72953200
C	-0.72855500	-0.34483000	0.03451600
O	-0.08362200	-1.10537200	-0.64836600
O	-6.98500400	-1.34998900	-0.49875300
C	-2.22429400	-0.35698600	0.09101100
C	-2.93430900	0.64911700	0.74708300
C	-4.32091300	0.61470800	0.76594900
C	-4.95721700	-0.43608900	0.12165500
C	-4.27658800	-1.44788500	-0.53999500
C	-2.89108100	-1.39840900	-0.55305200
N	-6.43043700	-0.47957700	0.14005700
O	-7.01966600	0.35679700	0.79324400
H	1.20551300	-1.10692200	1.86371900
H	2.66641800	-0.20296600	2.12468700
H	1.31364800	1.52317600	1.68353000
H	1.62969300	0.79086700	-1.25860000
H	3.35423600	2.36535600	0.70995500

H	3.24841700	2.79475400	-0.98188000
H	4.08074800	0.44084000	-1.57881000
H	5.17296100	1.65358700	-0.95549200
H	5.92912400	-0.16033900	0.14529700
H	4.48325200	-1.80758400	1.39271200
H	2.43235900	-1.02553900	-0.72270800
H	2.34253600	-2.38510900	0.36193400
H	-2.41017300	1.45991100	1.23600000
H	-4.89284700	1.38556400	1.26436000
H	-4.81422400	-2.24935600	-1.02811800
H	-2.32410400	-2.16951800	-1.05953700
H	5.38584100	1.38188700	1.71951000

TS3

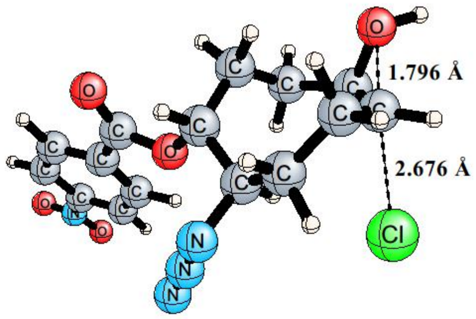

Zero-point correction= 0.325788 (Hartree/Particle)
Thermal correction to Energy= 0.348558
Thermal correction to Enthalpy= 0.349502
Thermal correction to Gibbs Free Energy= 0.269166
Sum of electronic and zero-point Energies= -1636.526147
Sum of electronic and thermal Energies= -1636.503376
Sum of electronic and thermal Enthalpies= -1636.502432
Sum of electronic and thermal Free Energies= -1636.582768
E= -1636.8519345 a.u., im. freq.= 405.62*i* cm^{-1}
0 1

C	-1.92848100	-1.83428300	0.40124700
C	-1.39072600	-1.02490600	-0.77652600
C	-2.10964600	0.28786500	-1.07621600
C	-3.54263700	0.15128400	-1.57337900
C	-4.57035100	-0.49592900	-0.63322600
C	-4.56908300	-0.07319200	0.80505200
C	-2.21859300	-1.00681800	1.65854900
O	-0.04190800	-0.60365500	-0.44010300
C	0.96621600	-1.42756700	-0.70145000
O	0.83780100	-2.51791300	-1.20795900
C	2.28619000	-0.85071100	-0.30091500
O	-4.61448100	-1.56941000	1.79813500
N	-1.38591100	0.98349700	-2.17812500
C	2.36614700	0.33785000	0.42578000
C	3.60671200	0.84306300	0.78699200

C	4.73697000	0.13697000	0.40271600
C	4.68639900	-1.04674200	-0.31955900
C	3.43983800	-1.54181800	-0.66968600
N	-0.43606300	1.67006200	-1.81599000
N	0.44398700	2.33001700	-1.58520200
N	6.05789800	0.66883600	0.78350900
O	7.04322200	0.03771900	0.46092000
O	6.09834700	1.71246700	1.40187700
H	-2.79950700	-2.41138500	0.08981400
H	-1.16193300	-2.57412300	0.64247300
H	-1.34405900	-1.63839200	-1.67808300
H	-2.08976100	0.92677200	-0.18989800
H	-3.54110100	-0.42811100	-2.50132600
H	-3.87917100	1.16002200	-1.81042500
H	-5.56191000	-0.28571100	-1.03510500
H	-4.45265900	-1.57979000	-0.64608200
H	-5.43533500	0.44334900	1.18993200
H	-1.53652500	-0.15400000	1.73261800
H	-2.02997700	-1.61752600	2.54545100
H	-5.26702800	-1.45985700	2.50953800
H	1.46692300	0.86546800	0.71576700
H	3.69339400	1.75993900	1.35399000
H	5.59410300	-1.56477400	-0.59773000
H	3.35935700	-2.46303200	-1.23312900
C	-3.61928000	-0.48330900	1.84438200
H	-3.72309100	0.09001100	2.76093400
Cl	-3.96900000	2.53294400	0.70449400

TS4

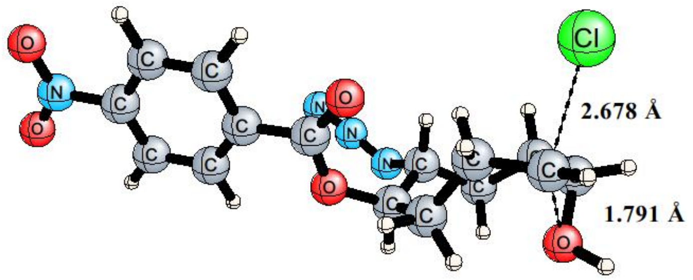

Zero-point correction= 0.325351 (Hartree/Particle)
Thermal correction to Energy= 0.348345
Thermal correction to Enthalpy= 0.349289
Thermal correction to Gibbs Free Energy= 0.268680
Sum of electronic and zero-point Energies= -1636.515048
Sum of electronic and thermal Energies= -1636.492054
Sum of electronic and thermal Enthalpies= -1636.491110
Sum of electronic and thermal Free Energies= -1636.571718
E= -1636.8403989 a.u., im. freq.= 400.99*i* cm^{-1}
0 1

C	1.93898900	-0.06716700	1.69855600

C	1.21550500	1.05666700	0.94246800
C	1.78601500	1.44796200	-0.42299900
C	3.22244100	1.98008200	-0.30182700
C	4.35659700	1.06019800	-0.77604100
C	4.95017400	0.08115800	0.21005000
C	4.19945200	-0.99933100	0.83156500
C	2.70702400	-1.11685500	0.86365400
O	-0.21935000	0.82334900	0.83572300
O	4.79180100	0.49935400	1.61403500
Cl	4.54345000	-2.74276700	-1.17128800
N	0.95440100	2.56025500	-0.97293000
N	-0.06752800	2.19503800	-1.54410800
N	-1.01687400	1.95024400	-2.09567700
C	-0.69544700	-0.26921300	0.24799500
O	-0.00724500	-1.14023300	-0.22872000
O	-6.90287200	-1.45669900	-0.44791400
C	-2.19139000	-0.29641900	0.22600400
C	-2.94619400	0.77584900	0.70297900
C	-4.33133300	0.72049600	0.65216200
C	-4.92087900	-0.41644800	0.11926700
C	-4.19500200	-1.49490900	-0.36525700
C	-2.81156500	-1.42517400	-0.30852800
N	-6.39249400	-0.48176400	0.06397100
O	-7.02436900	0.44209100	0.53352100
H	1.18009200	-0.58688500	2.28538600
H	2.60806100	0.39953800	2.41895300
H	1.23166700	1.95250200	1.56382900
H	1.74322000	0.61085600	-1.12435200
H	3.41250600	2.32237700	0.71952100
H	3.26980700	2.86804900	-0.93243100
H	4.05026000	0.48201200	-1.65391900
H	5.18031500	1.70011300	-1.09776800
H	5.98482600	-0.16492400	-0.00601200
H	4.79877700	-1.74243400	1.33856800
H	2.37812800	-1.10582500	-0.17386100
H	2.48003100	-2.10963100	1.24984900
H	-2.45862700	1.65214700	1.10956600
H	-4.93742200	1.53974500	1.01436700
H	-4.69692300	-2.36157200	-0.77353400
H	-2.20984300	-2.24628200	-0.67771400
H	5.59388400	0.28655700	2.11921100
